# Mitochondrial and Redox Modifications in Huntington Disease Induced Pluripotent Stem Cells Rescued by CRISPR/Cas9 CAGs Targeting

**DOI:** 10.3389/fcell.2020.576592

**Published:** 2020-09-22

**Authors:** Carla Lopes, Yang Tang, Sandra I. Anjo, Bruno Manadas, Isabel Onofre, Luís P. de Almeida, George Q. Daley, Thorsten M. Schlaeger, Ana Cristina Carvalho Rego

**Affiliations:** ^1^CNC-Center for Neuroscience and Cell Biology, University of Coimbra, Coimbra, Portugal; ^2^IIIUC-Institute for Interdisciplinary Research, University of Coimbra, Coimbra, Portugal; ^3^Division of Pediatric Hematology/Oncology, Children’s Hospital Boston, Boston, MA, United States; ^4^Harvard Stem Cell Institute, Boston, MA, United States; ^5^Faculty of Medicine, University of Coimbra, Coimbra, Portugal; ^6^Faculty of Pharmacy, University of Coimbra, Coimbra, Portugal; ^7^Howard Hughes Medical Institute, Boston, MA, United States; ^8^Department of Biological Chemistry and Molecular Pharmacology, Harvard Medical School, Boston, MA, United States

**Keywords:** induced pluripotent stem cells, huntington disease, mitochondrial dysfunction, neural stem cells, reactive oxygen species, transcriptional deregulation

## Abstract

Mitochondrial deregulation has gained increasing support as a pathological mechanism in Huntington’s disease (HD), a genetic-based neurodegenerative disorder caused by CAG expansion in the *HTT* gene. In this study, we thoroughly investigated mitochondrial-based mechanisms in HD patient-derived iPSC (HD-iPSC) and differentiated neural stem cells (NSC) *versus* control cells, as well as in cells subjected to CRISPR/Cas9-CAG repeat deletion. We analyzed mitochondrial morphology, function and biogenesis, linked to exosomal release of mitochondrial components, glycolytic flux, ATP generation and cellular redox status. Mitochondria in HD cells exhibited round shape and fragmented morphology. Functionally, HD-iPSC and HD-NSC displayed lower mitochondrial respiration, exosomal release of cytochrome c, decreased ATP/ADP, reduced PGC-1α and complex III subunit expression and activity, and were highly dependent on glycolysis, supported by pyruvate dehydrogenase (PDH) inactivation. HD-iPSC and HD-NSC mitochondria showed ATP synthase reversal and increased calcium retention. Enhanced mitochondrial reactive oxygen species (ROS) were also observed in HD-iPSC and HD-NSC, along with decreased UCP2 mRNA levels. CRISPR/Cas9-CAG repeat deletion in HD-iPSC and derived HD-NSC ameliorated mitochondrial phenotypes. Data attests for intricate metabolic and mitochondrial dysfunction linked to transcriptional deregulation as early events in HD pathogenesis, which are alleviated following CAG deletion.

## Introduction

Huntington’s disease is caused by CAG repeat expansion in the *HTT* gene, encoding for mHTT (No authors listed, 1993), with expanded polyglutamine (polyQ) stretch at the N-terminal. Symptoms include psychiatric disturbances, cognitive deficits and involuntary movements, correlated with a selective loss of striatal MSN and cortical atrophy (Roos, 2010). Neuropathological mechanisms described in HD include alterations in gene transcription, Ca^2+^ dyshomeostasis, metabolic and mitochondrial disturbances, and oxidative stress [reviewed in Gil and Rego (2008)]. Increased susceptibility of HD striatal cells to mitochondrial deregulation due to decreased Ca^2+^ handling, metabolic disturbances and mitochondrial ROS were previously documented by us (Oliveira et al., 2006; Ribeiro et al., 2014; Naia et al., 2017). Increasing evidence support that mitochondrial dysfunction occurs at HD early stages, e.g., as defined by proteomic analysis (McQuade et al., 2014); therefore, examining the mitochondrial processes that define the early stages of this complex human neurogenetic disease is of utmost importance.

Several groups have successfully generated HD patient-specific HD-iPSC that were differentiated into HD neural stem cells (HD-NSC) and neurons with striatal characteristics (Park et al., 2008; Zhang et al., 2010; Camnasio et al., 2012; Delli Carri et al., 2013; Mattis et al., 2015). Striatal-like MSN displayed altered electrophysiology, metabolism, cell adhesion and cell death for lines with long CAG, up to 180 repeats (Hd iPSC Consortium, 2012). Recently, the decreased bioenergetic capacity in HD-iPSC-derived striatal neurons was attributed to defects in glycolysis rather than mitochondrial defects, as it was reverted with pyruvate (Hd iPSC Consortium, 2020). Oxidative stress-related proteins, such as SOD1 (superoxide dismutase 1) and peroxiredoxin were also shown to be affected in HD-iPSC (Chae et al., 2012; Lu et al., 2014; Szlachcic et al., 2015).

The creation of isogenic lines in which expanded CAG was replaced by normal CAG repeat through homologous recombination (An et al., 2012), further enhanced by using CRISPR/Cas9 (An et al., 2014), has been also applied to HD. A 180 CAG iPSC was corrected using a CRISPR/Cas9 and piggyBac transposon-based approach, rescuing the phenotypic abnormalities (Xu et al., 2017). Another approach involved depletion of the *HTT* gene or allele-specific genome editing using Cas9 (Shin et al., 2016; Kolli et al., 2017; Monteys et al., 2017; Dabrowska et al., 2018), but none have specifically focused on mitochondrial-related abnormalities.

Here, we thoroughly investigated mitochondrial-based mechanisms in HD human iPSC and NSC early differentiated counterparts to identify mitochondrial abnormalities and altered metabolic pathways that may underlie neurodysfunction in HD and further investigated the influence of CAG repeat/exon 1 deletion in HD-iPSC using CRISPR/Cas9. Data indicate that mitochondrial dysfunction caused by deficient complex III and PDH activities are partially counterbalanced by glycolysis stimulation and leads to mitochondrial-driven ROS generation in early stages of differentiation in HD human cells. Importantly, these mitochondrial deficits are alleviated after the deletion of CAG expansion, reinforcing this strategy as an attractive HD therapy.

## Materials and Methods

### HiPSC Culture and Differentiation

Heterozygous human iPSC designated HD4-iPSC (XY, passages 4–30) with an expanded allele (72 CAG repeats) and a normal (19 CAG repeats) was generated by Park et al. (2008), while control AMS4-iPSC (XY, passages 7–30) was generated and characterized by de Almeida and collaborators (Onofre et al., 2016). Cells were maintained on a layer of mitotically inactivated murine embryonic fibroblasts (MEFs) for a variable number of passages or allowed to grow under feeder-free conditions on Matrigel^®^ and Geltrex^®^. Manual dissection was routinely used to passage the cells. MEFs were acquired from AMSBIO^®^ expanded for 3 passages and inactivated with mitomycin C.

iPSC were cultured in DMEM/F12 supplemented with 20% KSR, 2 mM glutamine, 1 mM non-essential amino acids, 1% penicillin/streptomycin (100 units/mL penicillin and 100 μg/mL streptomycin), 100 μM 2-mercaptoethanol and 10 ng/ml recombinant human FGF2. Cultures were fed daily and passaged at least once-a-week. When cells were grown in matrigel, medium was conditioned for 24 h in MEFs and filtered. Neural differentiation was based on dual SMAD inhibition with SB431542 (Lefty/Activin/transforming growth factor beta – TGFβ inhibitor), dorsomorphin (bone morphogenetic protein – BMP inhibitor) and XAV-939 (β-catenin-transcription inhibitor and axin stabilizing agent) (Chambers et al., 2009; Delli Carri et al., 2013; Nicoleau et al., 2013). Colonies were grown on 6-well plates in matrigel until reaching 100% confluence. Neural induction medium consisted of 1:1 mixture of two base media, DMEM/F12 and Neurobasal, 1% N2 (100x), 2 mM L-glutamine, 100 μM non-essential amino acids, 100 μM 2-mercaptoethanol, 1% penicillin/streptomycin and 2% B-27 (50x). Neural induction occurred between day 0 and day 10–12. For day 0 to day 5, cells were maintained in KSR medium without FGF2 and incubated with 5 μM dorsomorphin, 10 μM SB431542 and 1 μM XAV-939. Medium was changed every day. From day 5 to day 10–12, the medium was gradually replaced by 75% KSR + 25% N2 medium, 50% KSR + 50% N2 medium until reaching 100% N2 medium plus 5 μM dorsomorphin, 10 μM SB431542 and 1 μM XAV939 (Chambers et al., 2009; Delli Carri et al., 2013; Nicoleau et al., 2013). Between days 10 and 12, fields full of rosettes became morphologically visible. To allow the cells to differentiate, cells were replated in matrigel coated 12-well plates. For detaching, 500 μl of 1X Accutase^®^ was added in the plate and incubated at 37°C in 5% CO2, for 15–20 min. Accutase was diluted in DMEM/F12 medium pre-warmed at 37°C. Cells were collected and spun for 3 min at 1000 rpm, at room temperature (RT), and resuspended in 200 μl of media into a well of a 12-well plate. Cells were allowed to adhere for 30 min and then 300 μl N2 medium, supplemented with 10 μM Y-27632, 10 ng/ml FGF2 and 10 ng/ml EGF, was added. Dishes were carefully transferred at 37°C and left overnight in a 5% CO_2_ incubator. Cells were maintained in the same medium and passaged every 2–3 days for no more than 10 passages.

### Karyotype Analysis

The karyotype was assessed on metaphasic chromosomal spreads after GTG-banding performed at Centre of Genomics and Biotechnology of the University of Trás-os-Montes and Alto Douro (CGB-UTAD), Portugal.

### Transfection of CRISPR Into Human iPSC and PCR Characterization Following CRISPR Correction

Prior to transfection, hiPSC cells were exposed to 10 μM Y27632 for 1 h prior to collection, washed with PBS, dissociated into single cells using TryplE Select (4 min at 37°C), and washed with mTesR containing 10 μM Y27632. 1 million single-cell dissociated HD-iPSC were nucleofected with 10 μg dual sgRNA plasmid (sgRNA target sequences = GCCTCCGGGGACTGCCGTGC, gCAAACTC ACGGTCGGTGCAG) and 5 μg Cas9_GFP plasmid using the Lonza2D Nucleofector and the Amaxa Stem Cell Kit V (VCA-1003) with program B-016 as described previously (Burnight et al., 2017). Nucleofected cells were plated onto matrigel in a 12-well plate well using a 50:50 mix of fresh and iPSC conditioned mTesR medium with 10 μM Y27632. 44 h after transfection, the hiPSCs were again harvested with TryplE Select as before, and approximately 10,000 GFP+ cells sorted using a BD FACS Aria II (BSL2) were re-plated into a 6-well plate well in a 50:50 mix of fresh and iPSC conditioned mTesR medium with 10 μM Y27632 and grown in iPSC conditioned mTesR medium until colonies formed, after which the medium was switched to regular mTesR.

Each subclone was screened by genomic PCR. DNA was isolated by Qiagen DNeasy Blood and Tissue Kit according to the manufacturer’s instructions. The loci of interest were amplified by PCR (GoTaq Green Kit) using 7% DMSO for HTT amplification and without DMSO for YAP1, and cycling conditions were 90 s at 95°C, 40 × (30 s at 94°C; 30 s at 65°C; 90 s at 72°C). Primers for HTT primers were: 5′ GAGTCCCTCAAGTCCTTCCAGCA 3′, 5′ GCCCAAACTCACGGTCGGT 3′ (Jacquet et al., 2015) and primers for YAP1 were: 5′ TGAGTGATTTAAGGGTGAAA AATG 3′, 5′ TCACCATGTCCCAGTTTCTG 3′. PCR products were separated by gel electrophoresis using 1% agarose gels. HTT locus Sanger sequencing was done using PCR primers 5′ CCTCACCCCATTACAGTCTCACCAC 3′ and 5′ CACCACTTTACTTGGCAACCAC 3′, the Takara Primestar GXL PCR kit, 7% DMSO, cycling conditions 35 × (10 s at 98°C, 120 s at 68°C), and sequencing primer 5′ CAAGGGAAGACCCAAGTGAG 3′. Potential off-target sites were identified using CasOffFinder (pmid: 24463181) and then ranked according to the number and location of mismatches using a Python script. All identified potential off-target sites featured at least 3 mismatches, including at least 1 within the PAM-proximal 12 nucleotides.

### Transmission Electron Microscopy

For TEM, control and HD iPSC and NSC were collected and fixed with 2.5% glutaraldehyde in 0.1 M sodium cacodylate buffer and dehydrated in a graded ethanol series (70–100%). Following embedding in 2% molten agar, cell pellets were re-dehydrated in ethanol (30–100%), impregnated and included in Epoxy resin (Fluka Analytical). Ultrathin sections were mounted on copper grids and stained with lead citrate 0.2%, for 7 min. Observations were carried out on a FEI-Tec-nai G2 Spirit Bio Twin at 100 kV.

### Mitochondrial Labeling and Immunocytochemistry

Mitochondrial morphology was examined with pDsRed2-Mito. Cells were plated in a 24-well plate until 70% confluence; then transfection was performed according to the indicated procedures for Lipofectamine^TM^ 3000.

Cells were fixed with 4% Paraformaldehyde/PHEM (20 min, @RT), rehydrated with PBS/0.1% Triton X-100, blocked in 3% BSA/PBS (30 min) and incubated with primary antibody overnight at 4°C. Secondary antibodies and DAPI counterstain were applied for 1 h at room temperature. Primary antibodies are listed in Supplementary experimental procedures. Confocal analysis was performed on a Zeiss LSM 710 confocal system (Carl Zeiss Microscopy). For details see Supplementary Data.

### Immunoblotting

Cells were lysed in lysis buffer with protease inhibitor cocktail. For the isolation of nuclear and cytoplasmic fractions a Nuclear/Cytosol Fractionation Kit (BioVision, Inc.) was used according to manufacturer’s instructions. Protein lysates (25 μg) were denatured with SDS sample buffer at 95°C, for 5 min. Protein were loaded in 6% or 12% gel, subjected to SDS-PAGE and electrophoretically transferred onto polyvinylidene difluoride (PVDF) Hybond-P membranes. Immunoreactive bands were visualized with VersaDoc Imaging System (BioRad^®^, Hercules, CA, United States). For details see Supplementary Data.

### DNA and RNA Extraction, cDNA and RT-qPCR

Genomic DNA was extracted using PureLink^®^ Genomic DNA Kit and RNA with the PureZOL^®^ RNA Isolation Reagent. The purified DNA and RNA was then quantified with NanoDropR spectrophotometer Reverse transcription was performed with iScript^TM^ cDNA Synthesis Kit. Real-time PCR (qPCR) was performed with iQTM SYBR^®^ Green Supermix on a CFX96 Touch^TM^ Real-Time PCR Detection System. Q-PCR was performed according to manufacturer’s protocol. Tubulin and 18S were used for internal reference gene. Expression values were calculated using the 2^–ΔΔ*Ct*^ method. All PCR samples were run in technical triplicates, and the average Ct-values were used for calculations. The primers pairs are shown in Supplementary Data (Supplementary Table 2).

### Measurement of Adenine Nucleotides

In experiments aimed to inhibit glycolysis, the culture medium was replaced by DMEM with low glucose (2 mM) (GLUC) or supplemented with 17.5 mM 2-deoxy-D-glucose (2-DG). To inhibit mitochondrial ATP synthesis, 2 μg/ml oligomycin was added to glucose-containing medium or supplemented with 2-DG.

ATP, ADP, and AMP were measured by high-performance liquid chromatography (HPLC) following perchloric acid precipitation, as described previously (Stocchi et al., 1985). The chromatographic apparatus used was a Beckman-System Gold, consisting of a 126 Binary Pump Model and 166 Variable UV detector. Peak identity was determined by following the retention time of standards. Data normalized for total protein.

### Pyruvate Dehydrogenase E1α Subunit Protein Levels and Serine Phosphorylation

PDH expression and phosphorylation were assessed using PDH Enzyme Activity Microplate Assay Kit from MitoSciences (Oregon, United States).

### Mitochondrial Membrane Potential (ΔΨm) and Intracellular Ca^2+^ Measurements in Cell Population

ΔΨm was determined using the cationic fluorescent probe Rhodamine 123 (Rhod123). Briefly, iPSC were cultured in a 6-well plate and NSC in 96-well plate until reach confluence. For iPSC, detachment was required previously to incubation with the probes. Cells were incubated with accutase at 37°C in 5% CO2 for 15–20 min. Accutase was diluted in KSR medium pre-warmed at 37°C and left for 30 min to minimize the enzyme stress on cells. Then, iPSC were washed twice in Krebs medium, spunned and incubated at 37°C for 30 min with 8 μM Rhod123 and 1.5 μM Fura-2 acetoxy-methyl ester (Fura-2/AM). NSC were incubated in the 96-wells plates directly by following the same protocol, without enzymatic detach. After incubation, the basal fluorescence was taken in buffer with 8 μM Rhod123 during 5 min using a Microplate Spectrofluorometer Gemini EM (Molecular Devices, United States). Intracellular Ca^2+^ was measured with Fura-2/AM that has an excitation spectrum at 380 nm (calcium free) and 340 nm (calcium complex) (ratio 340/380) with emission at 540 nm. Oligomycin (2 μg/ml) and p-trifluoromethoxy carbonyl cyanide phenyl hydrazone (FCCP) (2 μM) (separately or together), were added to cells and the fluorescence was taken during another 5 min. Results were expressed as the difference between the increase in Rhod123 or Fura-2/AM fluorescence upon addition of oligomycin followed by FCCP or oligomycin plus FCCP and basal fluorescence values.

### Mitochondrial Superoxide Anion and Hydrogen Peroxide

The rate of mitochondrial superoxide production was measured using the mitochondria-specific probe MitoSOX Red (Life Technologies). iPSC and NSC were incubated with 5 μM MitoSOX and analyzed on a Microplate Spectrofluorometer Gemini EM. Cells were treated acutely with the stressor compounds (1 μM AA). NSCs were cultured for 24 h at 37°C in 96-well assay plates prior analysis. For NSC, Mitochondria peroxy yellow 1 (MitoPY1) basal levels were measured for 10–15 min followed by an acute stimulus with 3 μM myxothiazol. To measure extracellular H_2_O_2_ production, the Amplex Red Hydrogen Peroxide/Peroxidase Assay Kit was used. Cells were loaded with 10 μM AmplexRed reagent and 0.5 U/mL Horseradish (HRP) peroxidase. Fluorescence was measure for a total time of 40 min. Resultant fluorescence was analyzed on a Microplate Spectrofluorometer Gemini EM. The results were calculated as RFU per 500.000 cells for iPSC or per mg of protein for NSC. For details see Supplementary Data.

### Enzymatic Assays

For all enzymatic assays, cells were lysed and the resulting supernatant was used after protein quantification using the BioRad protein assay. SOD enzymatic activity was performed according to the SOD Assay Kit (Sigma-Aldrich). GPx and GRed activities and measurement of GSH and GSSG levels are detailed in Supplementary Data.

### Mitochondrial Respiratory Chain Complexes Activities

The mitochondrial-enriched fractions obtained from iPSC and NSC were assayed for the activity of mitochondrial complexes (Cx) I–IV by spectrophotometry. Detailed description of preparation of mitochondrial fractions and mitochondrial complexes activities in Supplementary Data.

### XF24 Extracellular Flux Analyzer

Mitochondrial respiration OCR, glycolysis ECAR and fatty acid oxidation measurements in iPSC and NSC was carried out using a Seahorse XF24 Extracellular Flux Analyzer (Seahorse Bioscience). Readings were normalized to the amount of protein and data analyzed using the Seahorse Wave software. Detailed description in Supplementary Data.

### Data Analysis and Statistics

Results are the mean ± SEM of the indicated number of independent experiments in figure legends. *F* test was performed to analyze the interaction term, as described in figure legends. At least three independent assays were performed for each experimental condition. Statistical significance was analyzed using parametric and non-parametric tests, namely one-way ANOVA and two-way ANOVA, followed by Bonferroni *post hoc* test, Student’s *t*-test for comparison between two Gaussian populations and Mann–Whitney and Kruskal–Wallis tests for non-Gaussian samples. *P* < 0.05 was considered significant.

## Results

### HD-iPSC and Neural Differentiated HD-NSC Express Mutant HTT and Display Cell-Specific Protein Expression Patterns

**FIGURE 1:**
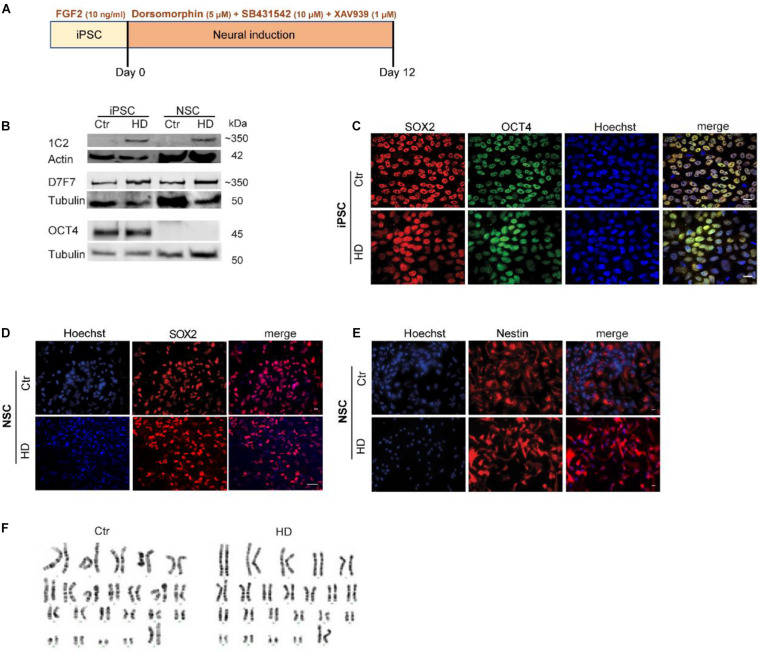
Characterization of HD and Control-iPSC and NSC Lines. **(A)** Scheme of neural differentiation protocol based on dual SMAD inhibition. **(B)** Western blots of HTT protein expression in iPSC and iPSC-derived NSC with the HTT antibody 1C2 (polyglutamine stretch) and D7F7 (residues surrounding Pro1220) demonstrate normal and mutant HTT expression, respectively. The pluripotency marker OCT4 is shown in iPSC only. **(C)** Expression of pluripotency markers OCT4 and SOX2 in iPSC. **(D,E)** Expression of neural stem cell markers SOX2 and Nestin, respectively. Scale bar (in panel **C,E**) indicates 50 μm. **(F)** Karyotyping and g-band analysis show that iPSC lines have a normal 46,XX karyotype.

NSC were generated from HD and control (Ctr) iPSC lines by a neural induction protocol for 12 days (Figure 1A; Delli Carri et al., 2013). HD-iPSC and HD-NSC express both normal and polyQ-expanded form of HTT (72 CAG) (Park et al., 2008; Figure 1B). iPSC pluripotency was confirmed by detection of OCT4 and SOX2 (Figures 1B,C). Successful differentiation into NSC was confirmed by the expression of SOX2 and nestin (Figures 1D,E). Furthermore, karyotyping and G-banding analysis showed that iPSC maintained a normal 46,XX karyotype (Figure 1F).

### Abnormal Mitochondrial Morphology in HD-iPSC and HD-NSC

**FIGURE 2:**
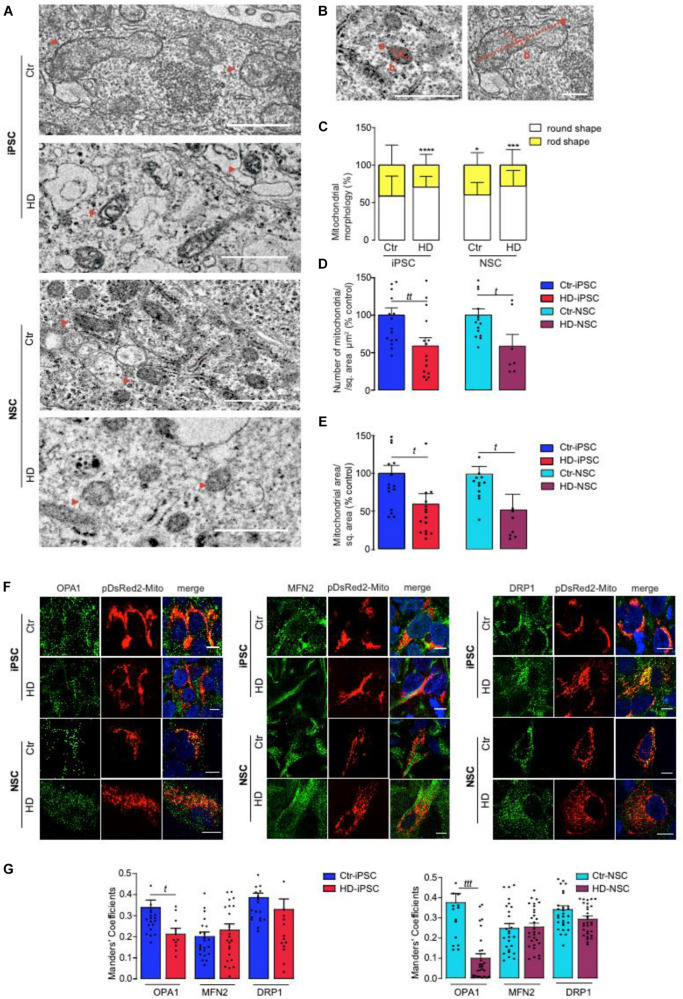
Mitochondrial Ultrastructure and Dynamics Abnormalities in HD and Control iPSC and NSC. **(A)** TEM analysis of mitochondrial morphology, size and number in control vs HD iPSC and NSC (red arrowheads point to some mitochondria). **(B–E)** Analysis of TEM images was made using JACoP ImageJ. Mitochondria were quantified as round shape when equal perpendicular axis is present (a,b) and with rod shape if one axis is longer (a > b) and normalized with cytoplasmatic area in each random field. Mitochondria number per region of interest (ROI) and mitochondria area (π.a.b) per region of interest (ROI) were analyzed. The evaluations were made in 18 random fields with the same magnification. Scale bar indicates 2000 nm. The results are expressed as the mean ± S.E.M. Statistical significance was determined using two-way ANOVA and Bonferroni multiple comparisons test. A significant effect of mitochondrial morphology (a,b) over genotype was observed [*F*(1,106) = 58.64, *p* < 0.0001]. ****p < 0.0001; ***p < 0.001; *p < 0.05. Student’s *t*-test: *t p* < 0.05, *tt p* < 0.01. **(F,G)** Immunocytofluorescence analysis of mitochondrial proteins OPA1, MFN2 (fusion) and DRP1 (fission) molecules in pDsRed2-Mito transfected cells. Images were photographed at ×63. The quantification of the images was performed on Z-stacks using JACoP ImageJ plugin. Scale bar of 10 μm. Values for three independent biological replicates, data presented as mean ± SEM. Student’s *t*-test *t*: *p* < 0.05; *ttt p* < 0.001.

Mitochondrial fragmentation has been previously associated with HD pathogenesis (Song et al., 2011). Thus, we first studied the ultrastructural abnormalities of mitochondria by TEM. Mitochondria were characterized as round shape if the axis (a and b) were equal, or rod shape if there was a tubular elongated morphology with a major and minor axis (Figures 2A,B). Although mitochondrial round shape was predominant in iPSC and NSC, the percentage of mitochondria with rod shape was significantly reduced in HD-iPSC and HD-NSC, when compared to Ctr cells (Figures 2A,C). HD-iPSC also exhibited a higher number of mitochondria with undeveloped cristae (Figure 2A). Both HD-iPSC and HD-NSC exhibited significantly lower (∼41%) number of mitochondria and smaller mitochondrial area *per* cytoplasmatic area analyzed, as compared to control cells (Figures 2D,E), demonstrating that mitochondria were smaller and less abundant, two features of immature organelle morphology. Thus, reduced number of mitochondria that retain round-shape with underdeveloped cristae characterize both HD-iPSC and HD-NSC.

Next we analyzed mitochondrial morphometrics by immunocytochemistry using pDsRed2-Mito to label mitochondria (Figures 2F,G). In general, Ctr-iPSC and NSC mitochondria assumed a more perinuclear and compact localization, which was less evident in HD iPSC and HD-NSC. Additionally, we observed a decrease in OPA1 co-localization with mitochondria in HD-iPSC and HD-NSC (Figures 2F,G). Data suggest diminished fusion in HD cells, in accordance with round-shaped mitochondria in HD cells.

### Extracellular Mitochondrial Components and Reduced Mitochondrial Respiratory Chain Activity in HD-iPSC and HD-NSC

Previously, multivesicles released from mesenchymal stem cells were reported to contain mitochondrial proteins and mtDNA (Phinney et al., 2015), suggesting that mitochondrial components can be secreted from cells in the form of extracellular vesicles, potentially affecting mitochondrial activity. In HD, several studies have demonstrated an imbalance toward fission events, leading to the accumulation of fragmented and damaged mitochondria (Cherubini et al., 2015, 2020). Considering the mitochondrial fragmentation observed in HD-iPSC and NSC we hypothesize that cells could shuttle mitochondrial proteins in exosomes. Therefore, exosomes were isolated from iPSC media (isolation of exosomes described in Supplementary Data) and analyzed by NanoSight and TEM demonstrating predominant cup-shaped membrane vesicles of ∼150 nm in diameter (Supplementary Figures S1A–C); similar results were observed for exosomes derived from NSC (not shown). Mass spectrometry analysis (described in Supplementary Data) showed that 31% of the proteomic content was common between iPSC and NSC (Supplementary Figure S1D). Several proteins are differentially released in exosomes from HD when compared to Ctr cells, with an enrichment in mitochondrial related functions and the presence of mitochondrial proteins in HD exosomes (Supplementary Figures S1E,F). In HD-iPSC, a 2-fold increase in proteins involved in apoptotic pathway (namely cytochrome C; *p* = 0.057) and ATP synthesis was observed, whilst in HD-NSC exosomal content included mitochondrial proteins involved in metabolic processes, namely ATP synthesis and TCA cycle (Supplementary Figure S1F). Increased exosomal release of metabolic-related proteins suggest a process by which mitochondria become dysfunctional.

**FIGURE 3:**
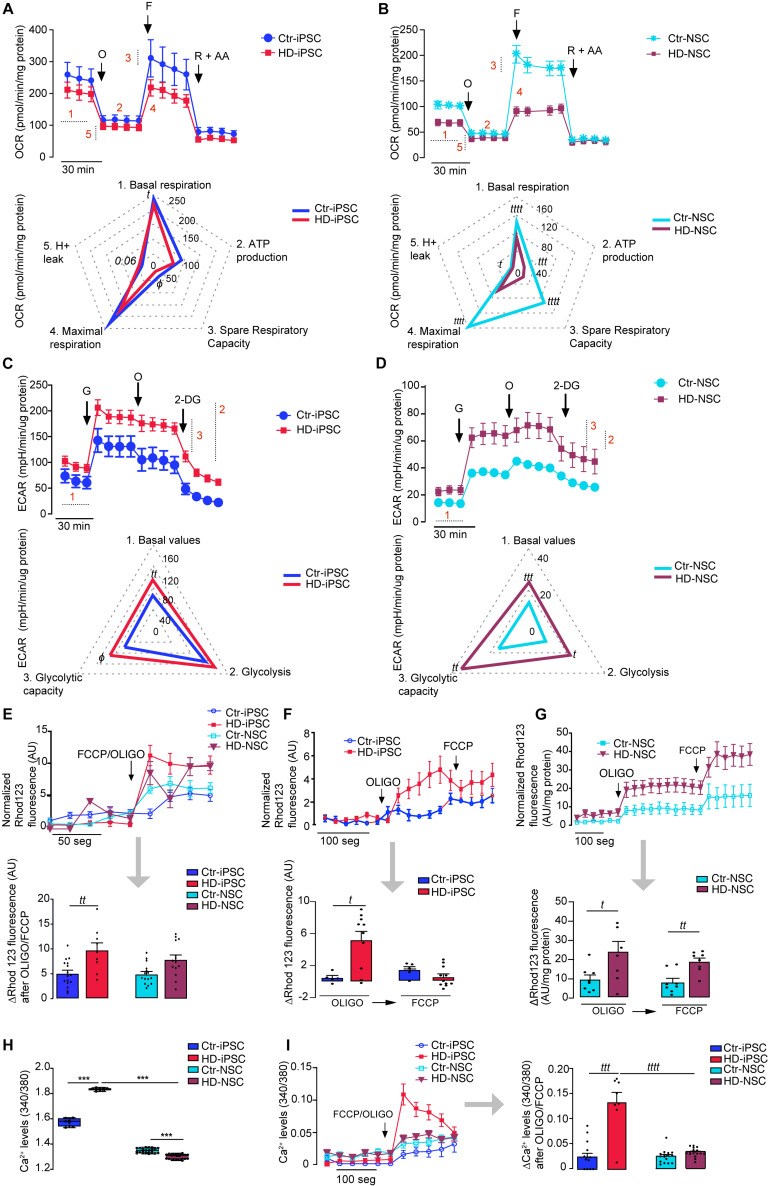
HD Promotes Glycolysis Stimulation and Abnormal High Mitochondrial Membrane Potential Plus Altered Calcium Homeostasis. **(A,B)** For analysis of mitochondrial OCR, inhibitors were injected sequentially: 1 μM oligomycin (O), 1 μM FCCP (F), 1 μM rotenone and 1 μM AA (R + A). OCR graphs shown on top and spider plots on the bottom: (1) basal respiration; (2) oxygen consumed for ATP generation through the complex V; (3) Spare respiratory capacity; (4) maximal respiration capacity; (5) passive proton leakage across the mitochondrial inner membrane. Values for mean ± S.E.M of at least three independent experiments. Student’s *t*-test: *t p* < 0.05; *tttt p* < 0.0001 or Mann–Whitney U test: *ϕ* < 0.05. **(C,D)** Analysis of ECAR. ECAR graphs shown on top and spider plots on the bottom: (1) Basal ECAR; (2) Glycolysis; (3) Glycolytic capacity. The results are expressed as the mean ± S.E.M of at least three independent experiments. Student’s *t*-test: *t p* < 0.05; *tt p* < 0.01, *ttt p* < 0.001 or Mann–Whitney U test: *ϕ* < 0.05. **(E–G)** Representative traces of mitochondrial membrane potential measured with the fluorescent lipophilic cationic probe Rhod123. Cells were exposed to 2 μg/ml oligomycin and 2 μM FCCP together or to 2 μg/ml oligomycin followed by 2 μM of FCCP. The bar graphs correspond to the variation of the fluorescence values; values for three independent experiments; Student’s *t*-test: *t p* < 0.05; *tt p* < 0.01. **(H,I)** Mitochondrial calcium release following exposure to oligomicyn plus FCCP and representative traces (as previous). The results are expressed as the mean ± S.E.M. from three independent experiments. Student’s *t*-test: (*t p* < 0.05; *tt p* < 0.01; *ttt p* < 0.001; *tttt p* < 0.0001) and one-way ANOVA, followed by Bonferroni *post hoc* test ***p < 0.001.

Therefore, we measured the OCR and the ECAR (Figures 3A–D). HD-iPSC (Figure 3A) and HD-NSC (Figure 3B) showed a significant decrease in basal respiration compared to Ctr cells. Oligomycin was used to determine ATP-linked OCR followed by FCCP to induce maximal respiratory capacity. Both parameters were slightly lower, whereas the proton leak was slightly higher (*p* = 0.06) in HD *versus* Ctr iPSC (Figure 3A). HD-iPSC also showed significantly reduced spare respiratory capacity (Figure 3A). Following neural differentiation, a marked decrease in basal OCR was observed in HD-NSC (Figure 3B). Other OXPHOS components, namely ATP-linked OCR, maximal respiratory capacity, proton leakage and spare respiratory capacity were significantly decreased in HD-NSC, when compared to Ctr-NSC (Figure 3B), evidencing reduced OXPHOS. These data suggest that HD mitochondria are less dependent on OXPHOS than control mitochondria and have lower biometabolic reserve in conditions of increased ATP demand.

Another energy production pathway is glycolysis. HD-iPSC have higher ECAR basal levels and glycolytic capacity, determined after glucose addition, indicating that HD cells rely more on glycolysis for energy production when compared to Ctr-iPSC (Figure 3C). HD-NSC also showed augmented basal proton production. After sequential addition of glucose to fuel glycolysis, an increase of 278% in ECAR was observed for HD-NSC, while for Ctr-NSC the increase was of 257% (Figure 3D). Indeed, HD-NSC exhibited increased dependence on glycolysis (Figure 3D). Accordingly, reduced OCR/ECAR ratio was observed in HD-iPSC and HD-NSC, supporting the decreased predominance of OXPHOS over glycolysis. After neural differentiation, OXPHOS and glycolysis decline as described previously (Birket et al., 2011). Data suggest that HD iPSC and NSC rely less on mitochondrial respiration and largely produce energy by glycolysis.

### Changes in Mitochondrial Transmembrane Potential and Calcium Dyshomeostasis in HD-iPSC and HD-NSC

Mitochondria not only regulate energy production, but also govern intracellular Ca^2+^ levels driven by the Ψm [for review, Demaurex et al. (2009)]. Thus, we estimated Ψm in HD-iPSC and HD-NSC by assessing their ability to retain rhodamine123. Exposure of HD-iPSC and HD-NSC to oligomycin and FCCP together, in order to achieve complete mitochondrial depolarization, caused a higher release of the probe when compared to Ctr cells, consistent with highly hyperpolarized mitochondria (Figure 3E).

Because hyperpolarized mitochondria may result from reversal of ATP synthase under conditions of inhibition of mitochondrial complex(es), we incubated oligomycin and FCCP at different time points. Results were consistent with oligomycin-evoked depolarization, which occurred in both HD and Ctr iPSC and differentiated counterparts, but was significantly more evident in HD-iPSC (Figure 3F) and HD-NSC (Figure 3G), when compared to control cells, indicating enhanced ATP synthase reversal in HD cells. In HD-iPSC oligomycin induced almost maximal release of the probe indicating that Ψm was largely secured through ATP synthase reversal.

Association between altered Ca^2+^ mitochondrial handling and Ψm abnormalities has been described in several studies, with mHTT showing a close interaction with mitochondria (Panov et al., 2002), although such interaction has not been unequivocally shown in HD-NSC (Supplementary Figure S2). Basal intracellular Ca^2+^ levels were slightly, but significantly, increased in HD-iPSC, but decreased upon differentiation into HD-NSC (Figure 3H). We assessed mitochondrial Ca^2+^ handling by challenging the cells with oligomycin plus FCCP to cause Ψm collapse. HD-iPSC mitochondria retained more Ca^2+^, compared to Ctr cells (Figure 3I), which could be related with higher Ψm (Figure 3E). Notably, the capacity to accumulate Ca^2+^ within the organelle was largely decreased in HD-NSC when compared to HD-iPSC (Figure 3I).

### Altered Mitochondrial Biogenesis and Complex III Activity in HD-iPSC and HD-NSC

**FIGURE 4:**
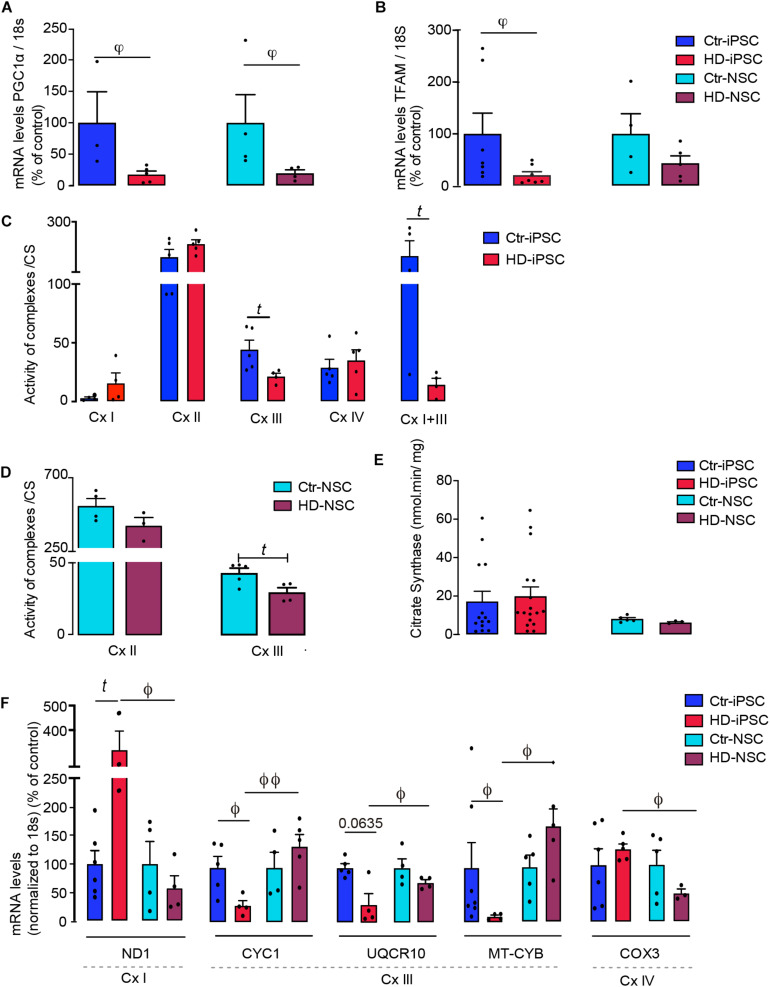
PGC-1α/TFAM Mediates Downregulation of Mitochondrial and Nuclear-Encoded Subunit Transcripts and Compromises Complex III Activity in HD. **(A,B)** mRNA levels of PGC-1α and TFAM normalized to 18S; values for five independent biological replicates shown as mean ± SEM; Kruskal–Wallis H test: φ < 0.05, φφ < 0.01. **(C–E)** Mitochondrial respiratory chain complexes (Cx) and citrate synthase (CS) activities determined in mitochondrial fractions of iPSC and NSC; values for five independent biological replicates shown as mean ± SEM; Student’s *t*-test: *t p* < 0.05. **(F,G)** mRNA levels of cytochrome c1 (CYC1), ubiquinol-cytochrome C reductase, complex III subunit X (UQCR10) and mitochondrial-encoded cytochrome B (MT-CYB); mitochondrial-encoded NADH:ubiquinone oxidoreductase core subunit 1 (ND1) and mitochondrial-encoded cytochrome C oxidase III (COX3), normalized to 18S. Values are the mean ± S.E.M. of five independent experiments, normalized for protein content and citrate synthase activity. Student’s *t*-test: *t p* < 0.05, Mann–Whitney U test ϕ < 0.05, ϕϕ < 0.01.

Peroxisome proliferator-activated receptor-γ coactivator α is a key component of mitochondrial biogenesis, which promotes expression of Mitochondrial Transcription Factor A (TFAM), involved in the synthesis of mitochondrial respiratory chain components. A pronounced reduction in PGC-1α mRNA levels was observed in both HD-iPSC and HD-NSC, whilst TFAM mRNA levels were decreased in HD-iPSC only, when compared to the respective control cells (Figures 4A,B).

To assess the influence of these results on mitochondrial function we measured the activities of mitochondrial complexes I to IV. We found that only Cx I + III activity was significantly reduced in HD-iPSC, mainly resulting from changes in the activity of Cx-III (Figure 4C). Concordantly, Cx-III activity was significantly decreased in HD-NSC (Figure 4D), while activity of citrate synthase was unchanged (Figure 4E). We further analyzed the mRNA levels of three genes encoding for Cx-III subunits: nuclear-encoded CYC1 and UQCR10 and mitochondrial-encoded MT-CYB; and mitochondrial-encoded ND1 for Cx-I and COX3 for Cx IV. Data revealed a reduction in the mRNA levels of Cx-III nuclear- and mitochondrial-encoded subunits, and an apparent compensatory increase in mRNA levels of Cx-I ND1 subunit in HD-iPSC, compared to Ctr cells (Figure 4F). In the case of HD-NSC, decreased Cx-III activity could not be attributed to changes in expression of these selected subunits, although we cannot exclude that other Cx-III subunits are affected. Interestingly, Cx-III CYC1, UQCR10 and MT-CYB subunits mRNA levels were increased in HD-NSC when compared to the pluripotent/undifferentiated counterparts (HD-iPSC), although reduced expression of Cx-I ND1 and Cx-IV COX3 subunits was observed in neural differentiated HD-NSC (Figure 4F).

The lower dependence on OXPHOS, accompanied by glycolysis stimulation in both HD-iPSC and HD-NSC is consistent with a decrease in mRNA levels of transcription factors involved in mitochondrial biogenesis and Cx-III (for HD-iPSC) subunits, leading to reduced enzymatic activity.

### Energetic Imbalance in HD-iPSC and HD-NSC

**FIGURE 5:**
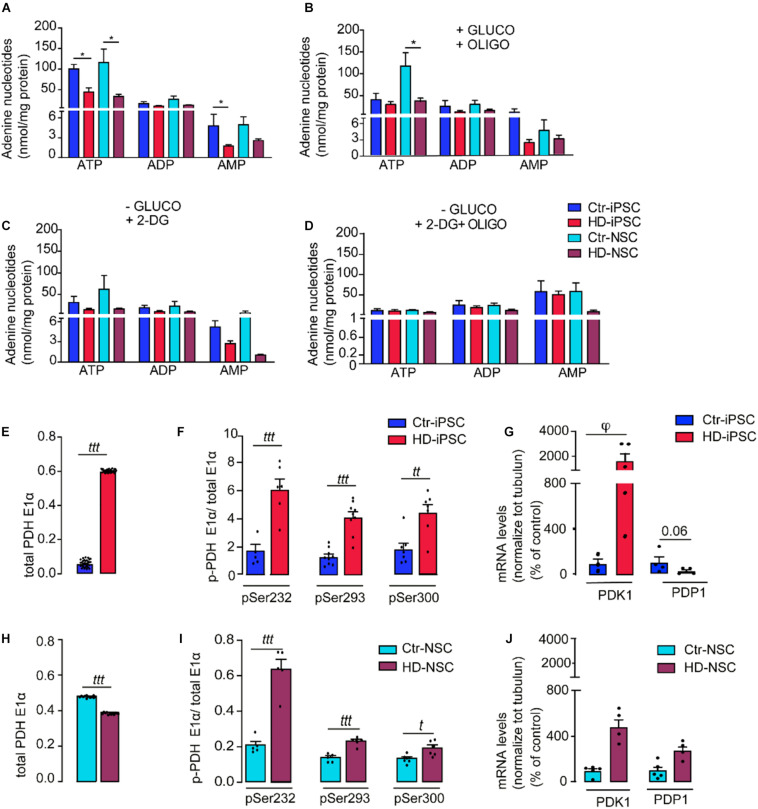
Energetic Imbalance in HD-iPSC and NSC. **(A)** The cell lysates were assayed for ATP, ADP, and AMP by separation in a reverse-phase HPLC. The results are expressed as the mean ± S.E.M of at least three independent samples; *p < 0.05 determined by one-way ANOVA and Bonferroni’s multiple comparisons test. **(B–D)** iPSC and NSC were challenged with 2 μg/ml oligomycin (OLIGO), or media depleted in glucose (-GLUCO) and supplemented with 2-DG or 2-DG plus 2 μg/ml oligomycin, for 2 h. Two way ANOVA analyses followed by Bonferroni’s multiple comparisons test revealed that there is a significant effect of treatment on ATP levels [*F*(4,74) = 8.76, *p* < 0.0001]. The results are expressed as the mean ± S.E.M of at least three independent samples; *p < 0.05; **(E,H)** Determination of total PDH levels in iPSC and NSC. **(F,I)** Quantification of the activity levels of phospho-PDH (Ser 232, 293, 300 of the E1-α subunit). Two way ANOVA analysis revealed that there is a significant effect of genotype on levels of phospho-PDH in iPSC [*F*(1,44) = 98.96, *p* < 0.001] and in NSC [*F*(2,26) = 32.58, *p* < 0.0001]. The results are expressed as the mean ± S.E.M of at least three independent samples. Student’s *t*-test: *ttt p* < 0.001; *tt p* < 0.001. **(G,J)** mRNA levels of PDK1 and PDP1 in iPSC and NSC; results are expressed as the mean ± S.E.M of at least three independent samples. Kruskal–Wallis H test φ < 0.05.

To examine the changes in bioenergetics in HD *versus* Ctr undifferentiated iPSC and differentiated NSC, we measured the levels of adenine nucleotides (ATP, ADP and AMP) before and after modulation of glycolytic and mitochondrial metabolic fluxes. Significantly lower levels of cellular ATP (Figure 5A) and ATP/ADP (not shown) were detected in HD-iPSC and HD-NSC, when compared to Ctr cells. In HD-iPSC decreased ATP was not counterbalanced by ADP or AMP (Figure 5A), suggesting the metabolic conversion into other metabolites that are part of the purine metabolic pathway. When adding oligomycin to the media, in the presence of glucose, to inhibit ATP synthase and stimulate the glycolytic flux (Figure 5B), a decrease in ATP levels was observed mainly in Ctr-iPSC (by 60%), compared to non-treated cells, confirming that Ctr-iPSC relies more on OXPHOS for ATP production than HD-iPSC (oligomycin repressed ATP levels by 36%); however, no changes occurred in NSC. After glycolysis inhibition with 2-DG all cells suffered a decrease in ATP levels, when compared to untreated cells, but HD-iPSC cells were more affected; addition of 2-DG markedly decreased ATP levels by 67% in HD-iPSC, and about 50% in Ctr-iPSC; in HD and Ctr NSC ATP decreased by 51% and 46%, respectively, (Figures 5A,C). Inhibiting both glycolysis and mitochondrial ATP generation with 2-DG plus oligomycin completed reduced ATP and ADP levels, elevating cellular AMP levels in Ctr- iPSC and NSC, and HD-iPSC, but not in HD-NSC (Figure 5D), suggesting severe metabolic defect.

### Enhanced Phosphorylation of E1 Subunit of Pyruvate Dehydrogenase Complex in HD-iPSC and HD-NSC

Because inhibition of PDH complex might contribute for decreased mitochondrial function in HD cells (Ferreira et al., 2011), we analyzed whether mitochondrial pyruvate metabolism through the PDH complex might be affected in HD iPSC and NSC. Phosphorylation of PDHE1α by PDK 1–4 causes PDH inhibition, while dephosphorylation by PDP 1–2 promotes its activation. The protein levels of PDHE1α subunit were significantly increased in HD-iPSC (Figure 5E) but decreased after neural differentiation (Figure 5H). High levels of phosphorylated PDHE1α (Ser 232, 293 and 300) were found in both HD iPSC and NSC, underlying PDH partial inactivation (Figures 5F,I). We also measured PDK1 and PDP1 mRNA levels. Only PDK1 can phosphorylate all 3 serines (Korotchkina and Patel, 2001) and an increase in gene expression for PDK1 was previously detected in stem cells (Varum et al., 2011), while PDP1 can dephosphorylate all three sites with similar preference (Rardin et al., 2009). PDK1 mRNA levels were upregulated in HD-iPSC, whereas PDP1 mRNA levels were downregulated (Figure 5G), correlating with enhanced PDHE1 phosphorylation. In HD-NSC, PDK1 levels were also increased (Figure 5J), although in a less extent when compared to HD-iPSC (Figure 5G). Contrarily to iPSC, HD-NSC displayed slightly augmented PDP1 mRNA levels (Figure 5J), which may explain the relative reduction in all Ser phosphorylation in NSC (Figure 5I).

These data indicate that, apart from decreased Cx-III activity, dysfunctional mitochondrial metabolism is due to PDH complex inactivation in undifferentiated HD iPSC and NSC.

### HD-iPSC and HD-NSC Exhibit Increased Levels of Mitochondrial and Cellular ROS and Dysregulation of Antioxidant Response

**FIGURE 6:**
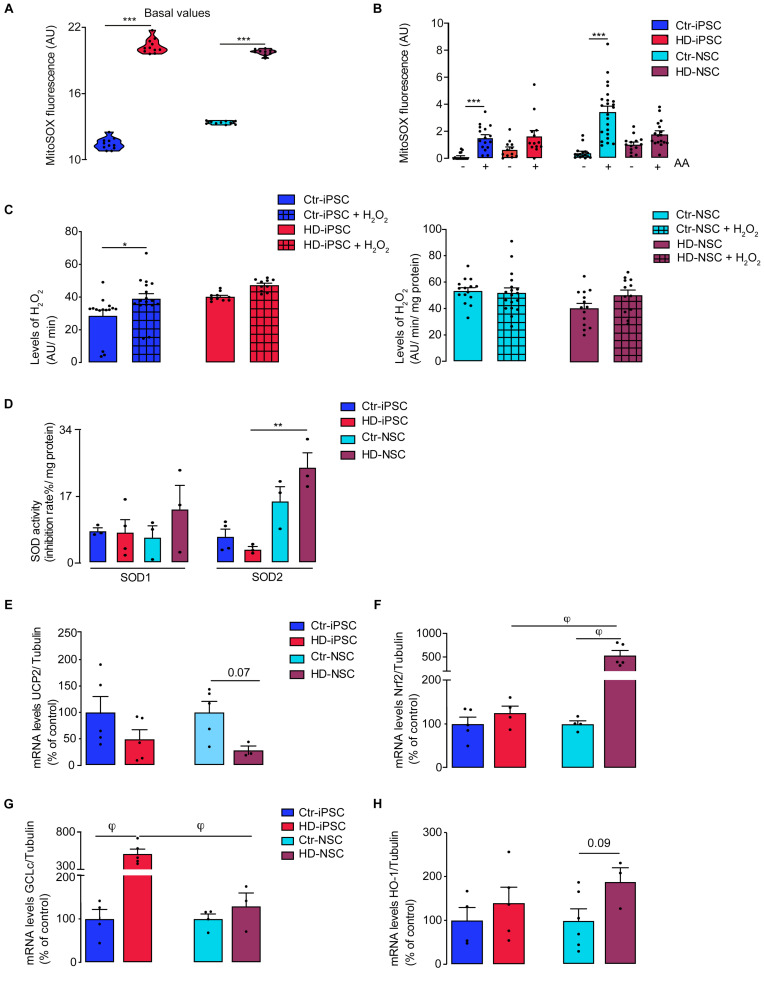
Increased Vulnerability to Oxidative Stress in HD Cells Associated to a Reduced Antioxidant Response. **(A,B)** Basal levels of mitochondrial O_2_^∙–^ and generation of O2^–^ after acute exposure to 1 μM AA in iPSC and NSC; *n* = 3 independent biological replicates; values shown as median with interquartile range for A and or mean ± SEM for B; ***p < 0.001, one way ANOVA and Bonferroni’s multiple comparisons test. **(C)** Detection of extracellular hydrogen peroxide by AmplexRed assay in iPSC and NSC; *n* = 3 independent biological replicates; values shown as mean ± SEM; one-way ANOVA and Bonferroni’s multiple comparisons test *p < 0.05. **(D)** Superoxide dismutase (SOD) activity were determined in iPSC and NSC. *n* = 3 independent biological replicates; values shown as mean ± SEM; one-way ANOVA **p < 0.01. **(E–H)** mRNA expression of UCP2, Nrf2, GCLc and HO-1 normalized to tubulin. Results are the mean ± SEM of at least 3 independent samples. Kruskal-Wallis H test φ < 0.05.

Because inhibition of Cx-III is linked to electron leakage at the mitochondrial respiratory chain and increased production of O_2_^⋅–^, we further determined the levels of mitochondrial ROS in HD iPSC and NSC. Both HD cells exhibited higher basal levels of mitochondrial O_2_^⋅–^ (Figure 6A) as well as increased production of ROS after addition of AA (Figure 6B). Likewise, HD-iPSC produced increased levels of cellular H_2_O_2_ under basal conditions, which were exacerbated after pre-incubation with oxidant stimulus (Figure 6C).

Considering that ROS production is influenced by the activity of endogenous antioxidant enzymes, and O_2_^⋅–^ dismutation is the main source of H_2_O_2_, we evaluated the activity of SOD1 and SOD2, as both may co-exist in mitochondria. Apart from a significant increase in SOD2 activity in HD cells upon neural differentiation (Figure 6D), which may partially counterbalance the enhanced production of mitochondrial O_2_^⋅–^, no other significant differences were detected in SODs levels (including acetyl-SOD2 at Lys68) (2A-B) or total SOD activity (not shown). Another mechanism involved in attenuating ROS production and metabolism regulation is mediated by the protein UCP2. Lower UCP2 levels facilitate ROS accumulation, which seems to contribute for differentiation into certain lineages (Zhang et al., 2011). Our results show a pronounced downregulation of UCP2 mRNA levels in HD-NSC compared to controls (*p* = 0.07) (Figure 6E), supporting mitochondrial-driven oxidative stress.

Increased levels of ROS can induce the activation of the Nrf2, a transcription factor that regulates the antioxidant response by activating phase II detoxification enzymes, which are described to be compromised in HD NSC (Quinti et al., 2017). In our results, HD-NSC only showed significantly higher levels of Nrf2 mRNA when compared to HD-iPSC and Ctr-NSC (Figure 6F); an increase in P(Ser40)Nrf2 in cytoplasm was observed in HD-iPSC (Supplementary Figure S3C), supporting the increase in ROS levels. To further study the impact of Nrf2/ARE pathway on antioxidant gene expression we analyzed GCLc and HO-1 transcripts (Figures 6G,H), the latter supporting GSH synthesis. GCLc mRNA levels were significantly upregulated in HD-iPSC and, interestingly, a pronounced reduction was observed after differentiation (Figure 6G). HD-iPSC had higher levels of GSH (Supplementary Figure S3D), suggesting that glutathione system is important for the maintenance of the redox state.

### CRISPR/Cas9-Targeted Deletion of Exon-1/CAG Repeats Reverses HD-Related Phenotypic Abnormalities

**FIGURE 7:**
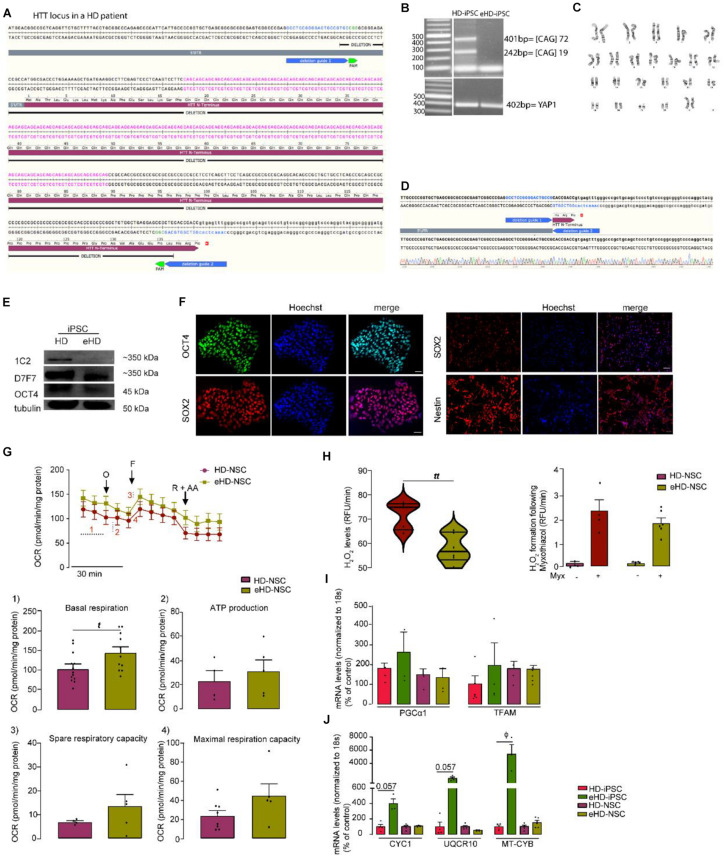
CRISPR/Cas9-mediated Deletion of the polyQ-encoding Region in HTT Gene in HD-iPSC Reverses the Phenotypic Abnormalities. **(A)** Schematic overview of mutant HTT cutting strategy. **(B)** Overview of a PCR for a successfully targeted iPSC clone. **(C)** Karyotype analysis of corrected HD-iPSC. **(D)** Sanger sequencing performed for the PCR bands cut from the gel. **(E)** Expression of normal HTT is maintained in iPSC, as defined using HTT antibody 1C2 (polyglutamine stretch) and D7F7 (residues surrounding Pro1220). **(F)** The CAG deleted iPSC maintains pluripotency as shown by positive immunostaining for the pluripotency marker OCT4 and the potential to differentiate into NSC by positive immunostaining for Nestin. **(G)** Measures of mitochondrial respiration in HD-NSC and isogenic control (. **(H)** Basal levels of mitochondrial H_2_O_2_ and generation of H_2_O_2_ after acute exposure to 3 μM myxothiazol (Myx) in control, HD and CAG deleted NSC; values are the median with interquartile range (violin plot) or mean ± SEM (bar graph) of at least three independent samples. *tt p* < 0.01; Student *t*-test. **(I,J)** mRNA levels of PGC-1α and TFAM, cytochrome c1 (CYC1), ubiquinol-cytochrome C reductase, complex III subunit X (UQCR10) and mitochondrial-encoded cytochrome B (MT-CYB) for HD-iPSC and NSC and CAG deleted controls; values for four independent biological replicates shown as mean ± SEM; ϕ < 0.05, Mann–Whitney U test.

We employed a CRISPR/Cas9 excision strategy to remove the expansion of CAG repeats in exon 1 of the HTT gene. We designed a pair of HTT sgRNAs for excision with S. pyogenes Cas9 to specifically excise the repeat-containing exon 1 fragment (Figure 7A). The sgRNA pair was expressed from a plasmid containing two human Pol III U6 promoter driven sgRNA expression cassettes. The sgRNA plasmid and a Cas9-GFP expression plasmid (pCas9_GFP, a gift from Kiran Musunuru, Addgene #44719) were co-transfected into hiPSCs as described previously (Burnight et al., 2017). The transfected cells were sorted 2 days later to enrich for Cas9-GFP high-expressing cells followed by single-cell plating, colony picking, expansion, and analysis. Targeted clones were identified by PCR (Figure 7B). Additionally, karyotyping and G-banding analysis showed that CRISPR targeted cells maintained a normal 46,XX karyotype (Figure 7C). Sanger sequencing confirmed the precise excision of the region between the two expected Cas9-induced double-strand breaks (Figure 7D). Potential off-target sites were identified using CasOffFinder. Consistent with published observations according to which off-target mutations at such sites are exceedingly rare (pmid: 25425480, 24996165, 26212079, 24996167) we did not find any *de novo* mutations among the four tested most-likely off-target sites (genomic DNA PCR product Sanger sequencing data shown for the highest-scoring off-target loci of the 5′ and 3′ HTT sgRNAs) (Supplementary Figure S4). Successful deletion of the CAG expansion was verified by western blotting using antibodies for polyglutamine stretch (1C2) and HTT (residues surrounding Pro1220) (Figure 7E). We then examined the pluripotency characteristics of the excised iPSC (eHD-iPSC) showing a positive staining for OCT4 and SOX2, and for Nestin after differentiation into NSC (eHD-NSC) (Figure 7F). Next, we sought to establish whether CAG deletion results in reversal of previously observed HD key metabolic phenotypes, namely mitochondrial respiration, mitochondrial driven H_2_O_2_ production and expression of genes related to mitochondrial biogenesis or bioenergetics. We found that CAG excision results in improved basal respiration (Figure 7G) and a significant reduction of mitochondrial ROS levels in eHD-NSC (Figure 7H). Furthermore, while no changes were observed in PGC-1α or TFAM mRNA levels (Figure 7I), involved in mitochondrial biogenesis, in eHD-iPSC or eHD-NSC, a significant increase in mRNA levels of complex III subunits (CYC1, UQCR10 and MT-CYB) was observed in eHD-iPSC (Figure 7J), supporting the rescue in mitochondrial function.

## Discussion

In this study, we observed that early differentiated human HD iPSC and NSC exhibit intricate features of mitochondrial and metabolic impairment linked to decreased activities of complex III and PDH complex, reduced oxygen consumption and mitochondrial ATP production, with the organelle exhibiting enhanced production of mitochondrial-driven ROS and a fragmented morphology. Of relevance, altered mRNA levels of nuclear-encoded complex III subunits and PDK *versus* PDH phosphatase endorse inhibitory effects on respiratory chain complex III and PDH. These changes were accompanied by hyperpolarized mitochondria that retained more calcium and by glycolysis stimulation to partially compensate the cellular bioenergetic demand imposed by mHTT expression. A link between nuclear morphology and enhanced store-operated calcium channels activity was described in HD iPSCs-derived neurons, supporting the previous findings of calcium transport deregulation in HD models (Nekrasov et al., 2016).

Mitochondrial fission and fusion proteins regulate morphology, function, integrity and topographic distribution of mitochondria. Indeed, HD models exhibit altered expression of DRP1, Fis1, OPA1 and MFN1/2 proteins (Song et al., 2011; Shirendeb et al., 2012). Concordantly, we showed abnormal mitochondrial dynamics in HD iPSC and NSC, in which the organelle assumes a spherical morphology (Facucho-Oliveira et al., 2007; Prigione et al., 2010; Zhang et al., 2011; Kelly et al., 2013) and lower mitochondrial levels of OPA1 in HD-iPSC and NSC, which may underlie altered mitochondrial morphology (Chen et al., 2016).

Our result is supported by the observation that mHtt reduced the expression of OPA1 mRNA in R6/2 mice and *postmortem* HD patient’s brains (Shirendeb et al., 2011; Hering et al., 2017). The interaction of mHTT with DRP1 increases its GTPase activity resulting in fragmented mitochondria. Consequently, Drp1/Fis1-mediated mitochondrial fission has been described as a major player in the progression of HD (Guo et al., 2013; Joshi et al., 2019). In our study the levels of DRP1 were similar to controls, suggesting that its upregulation could be associated to late-stage HD progression (Shirendeb et al., 2011).

Alongside, we observed alterations in mitochondrial respiration and bioenergetics in HD iPSC and NSC, as described in higher CAG length cell lines (Hd iPSC Consortium, 2012). These findings are in agreement with reduced gene expression of two nuclear-encoded and one mitochondrial-encoded subunits of mitochondrial Cx-III (CYC1, UQCR10 and MT-CYB) and Cx-III activity in human HD-iPSC. Importantly, we observed that exosomes can package mitochondrial proteins (e.g., cytochrome c), which increased release from HD cells might constitute a mechanism that relates with decreased Cx-III subunit expression and activity; although its physiological relevance is still unclear, exosomal release of mitochondrial components may constitute a cell survival mechanism in response to oxidative stress and mitochondrial dysfunction (Phinney et al., 2015).

Different lines of evidence indicate that in iPSC Ψm appears to be maintained by glycolytic ATP, used for maintaining the hydrolase activity of complex V (ATP synthase) and the higher Ψm. Consistently, previous studies showed that ATP synthase can be reversed in iPSC, hydrolyzing glycolytic ATP to maintain the Ψm and mitochondria in a less functional state resorting to the inhibition of respiratory chain complex(es) (Cho et al., 2006; Nicholls, 2006; Zhang et al., 2011; Lorenz et al., 2017; Ghosh et al., 2020). Here, we show that this is exacerbated in HD iPSC and NSC as displayed by mitochondrial hyperpolarized status. In a recent study, NSC expressing *HTT* exon 1 with expanded 71 and 122 CAG repeats displayed impaired activities of complex I and II + III, increased retention of TMRM, suggesting hyperpolarized mitochondria, associated to altered morphology (Ghosh et al., 2020). Importantly these findings highlight that exon 1 *HTT* fragments are sufficient to cause mitochondrial dysfunction in a CAG number dependent manner supporting its role in the pathogenesis of HD.

A key metabolic regulator favoring glycolysis *versus* OXPHOS involves the PDH complex (Varum et al., 2011). hESC express high levels of hexokinase2 (localizing to the outer mitochondrial membrane) and have an inactive PDH complex. Also, mRNA levels of PDK1,3 declined significantly during neuronal differentiation, whereas PDP1 mRNA levels increased, favoring PDH activity in neurons (Zheng et al., 2016). Here we show that PDH E1α subunit significantly increased in HD-iPSC, but serines 232, 293 and 300 residues were highly phosphorylated in both HD iPSC and NSC, indicating decreased activity of this enzymatic complex; indeed, PDK1 mRNA levels were upregulated and PDP1 downregulated in HD iPSC, consistent with enhanced PDHE1α phosphorylation, as identified in distinct HD models and patient’s brain tissue (Sorbi et al., 1983; Ferreira et al., 2011; Naia et al., 2017). Indeed, several studies reported, in human iPSC and differentiated cell, mitochondrial dysfunction and metabolic deficits attributed to mHTT (An et al., 2012; Hd iPSC Consortium, 2012, 2017, 2020; Xu et al., 2017; Ghosh et al., 2020) but to our knowledge PDH activity was not assessed. Our results point to decreased PDH activity as an early event in HD pathophysiology.

Antioxidant genes such as UCP2 are expected to be upregulated in iPSC and reduced UCP2 expression facilitates ROS accumulation in hiPSC and hESC (Zhang et al., 2011). Consistently, we observed a decrease in UCP2 mRNA levels in HD-iPSC (67%), suggesting that UCP2 can assume an important role in endogenous ROS management, prompting HD-iPSC susceptibility to mitochondrial dysfunction associated with increased ROS production. A rise in mitochondrial O_2_^⋅–^ levels was consistent with Cx-III inhibition. Previous studies described increased cell death in striatal-like neurons differentiated from HD-iPSC lines in response to toxic stressors, (Hd iPSC Consortium, 2012) and increased generation of mitochondrial ROS in HD mouse striatal cells (Ribeiro et al., 2014). Moreover, proteomic analysis in the same 72 CAG HD-iPSC used in this work showed that antioxidant enzymes, such as SOD1 or peroxiredoxin (Prx) were downregulated or up-regulated, respectively, (Chae et al., 2012). While we observed mitochondrial abnormalities, other researchers reported the absence of mitochondrial dysfunction, claiming that bioenergetic deficits and ROS production are not decisive factors in HD pathology in the pre-symptomatic stage despite recognizing the involvement at later stages of the disease (Hamilton et al., 2020). The reason for this discrepancy is not clear but could be attributed to differences in cell culture and/or methodologies.

A question arising from this work is the variability of the iPSC genetic background, which may result in inaccurate interpretation of disease phenotypes *in vitro.* Several approaches have been described to block mHTT expression and therefore clarify the role of CAG expansion on disease phenotype and potential use as therapeutic strategy. In this work, we demonstrate that CRISPR-Cas9-mediated excision of exon 1 fragment-containing CAG repeats in HD-iPSC (eHD-iPSC) can reverse phenotypic alterations found in HD iPSC and NSC, including deficits in mitochondrial respiration, ROS production and mitochondrial gene-related expression further demonstrating that HD-associated mitochondrial and metabolic impairments are associated to the HD genotype and not to variability of the iPSC genetic background, reprograming or differentiation protocols.

Genetic correction of HD-iPSC using CRISPR/Cas9-assisted techniques was previously achieved showing rescuing effects (An et al., 2014; Xu et al., 2017), but not thoroughly with a focus on mitochondria as in the present work. Cellular abnormalities including mitochondrial deficits, low levels of BDNF, altered cadherin and TGF-β signaling, impaired neural rosette formation and increased susceptibility to growth factor withdrawal were rescued in corrected isogenic HD lines (An et al., 2014; Xu et al., 2017). Other approaches used non-allele selective suppression or SNP-based approach to efficiently disrupt exogenous/endogenous WT/mutant *HTT* gene, being associated with decreased mHTT aggregation (Shin et al., 2016; Kolli et al., 2017; Monteys et al., 2017; Yang et al., 2017). These and our results support gene-silencing approaches such as the recently reported intrathecal administration of antisense oligonucleotide designed to inhibit *HTT* transcripts, which resulted in dose-dependent reduction in mHTT levels in human HD carriers (Tabrizi et al., 2019); it will be relevant to examine whether these novel gene silencing approaches also impact on mitochondrial function and redox activity in HD patient’s cells, along with major HD-related symptoms.

## Conclusion

In summary, our study shows in large detail mitochondrial impairment that could be attributed to reduced mitochondrial biogenesis and decreased CxIII and PDK1 levels, causing metabolic imbalance, and enhanced mitochondrial generation of ROS, linked to round shape mitochondrial morphology in early stages of undifferentiation, in human HD iPSC, which were mostly replicated in iPSC-derived NSC expressing mHTT. Of relevance, CAG repeat excision in the mutant *HTT* gene ameliorated relevant mitochondrial-associated phenotypes, including the expression of mitochondrial and nuclear-encoded Cx-III subunits, demonstrating a positive impact of CAG correction strategies on early onset HD phenotypes related with mitochondrial deregulation (Figure S5_Graphical abstract).

## Data Availability Statement

The raw data supporting the conclusions of this article will be made available by the authors, without undue reservation.

## Ethics Statement

There are no ethical concerns since both heterozygous (19/72 CAG repeats) human HD and control iPSC have been previously generated, at GD (Harvard Medical School, Boston, MA, United States) and LA (University of Coimbra, Coimbra, Portugal) labs, respectively (Park et al., 2008; Onofre et al., 2016); HD-NSC and control NSC, as well as CRISPR/Cas9 iPSC and NSC corrected lines were obtained from original iPSC.

## Author Contributions

CL and ACR were responsible for the experimental design, data interpretation and writing of the manuscript. CL performed and analyzed most of the experiments and prepared the initial draft of the manuscript. YT and TS were responsible for CRISPR/Cas9 correction and assay design. SA and BM performed the MS analysis. ACR supervised the study and provided funds. All co-authors reviewed and edited the manuscript.

## Conflict of Interest

The authors declare that the research was conducted in the absence of any commercial or financial relationships that could be construed as a potential conflict of interest.

## Abbreviations

Ψ m: mitochondrial transmembrane potential
AA: antimycin A
Ctr: control
ECAR: extracellular acidification rate
HD: huntington’s disease
iPSC: induced pluripotent stem cells
mHTT: mutant huntingtin
MSN: medium spiny neurons
Nrf2: nuclear factor erythroid derived 2-related
NSC: neural stem cells
OCR: oxygen consumption rate
PDH: pyruvate dehydrogenase
PDK: pyruvate dehydrogenase kinases
PDP: PDH phosphatases
PGC1 α: peroxisome proliferator-activated receptor- γ coactivator α
polyQ: expanded polyglutamine
Prx: peroxiredoxin
ROS: reactive oxygen species
SNP: single nucleotide polymorphisms
SOD: superoxide dismutase
TEM: transmission electron microscopy
TFAM: mitochondrial transcription factor.
