# Supplementary material for: Mitochondrial and Redox Modifications in Huntington Disease Induced Pluripotent Stem Cells Rescued by CRISPR/Cas9 CAGs Targeting
Source: Front Cell Dev Biol. 2020 Sep 22;8:576592. doi: 10.3389/fcell.2020.576592 (PMC7536317; doi:10.3389/fcell.2020.576592)
Supplement: Supplementary file 1 [file Data_Sheet_1.pdf]

Supplemental Material

Supplementary Figures

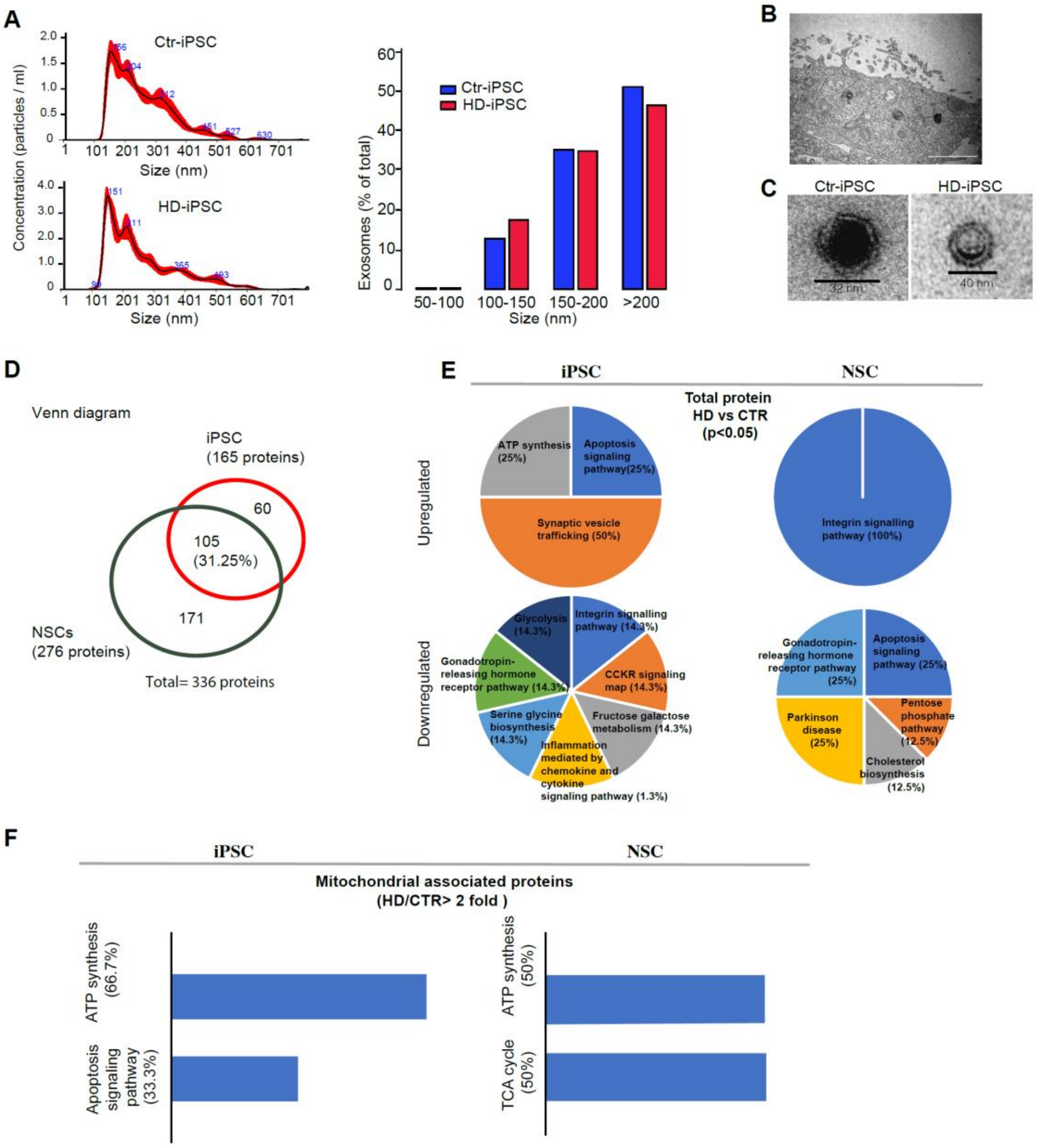

Figure S1. Characterization of iPSC and NSC Exosomes. (**A**) Size distribution of exosomes derived from iPSC measured from Nanosight. (**B**) Exosomes released from iPSC. (**C**) Exosomes isolated from iPSC media visualized by transmission electron microscopy. (**D-F**) Mass spectrometry analysis for exosomal proteins. (**D**) Venn diagram comparison of exosomal proteomes of HD iPSC and NSC. (**E**) GO enrichment analysis considering cellular pathways of the exosomal proteins altered in HD vs Ctr iPSC and NSC. (**F**) GO analysis of cellular pathways specific associated to exosomal mitochondrial proteins.

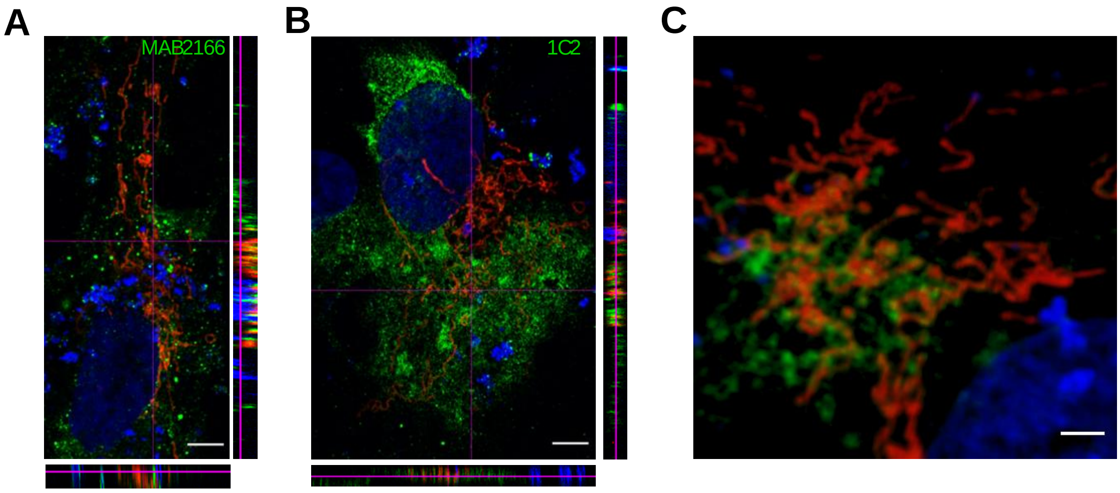

Figure S2. Immunocytofluorescence analysis of **(A)** huntingtin (MAB2166- a.a. 181-810) and **(B)** polyglutamine expansion present in mutant huntingtin (1C2) in pDsRed2-Mito transfected HD-NSC cells. Single plane and orthogonal views of fluorescent confocal Z stacks. (**C**) Full 3D imaging (3D viewer plugin) of the same mitochondrial population shown in panel B. Images were taken at ×63. Scale bar of 5 µm.

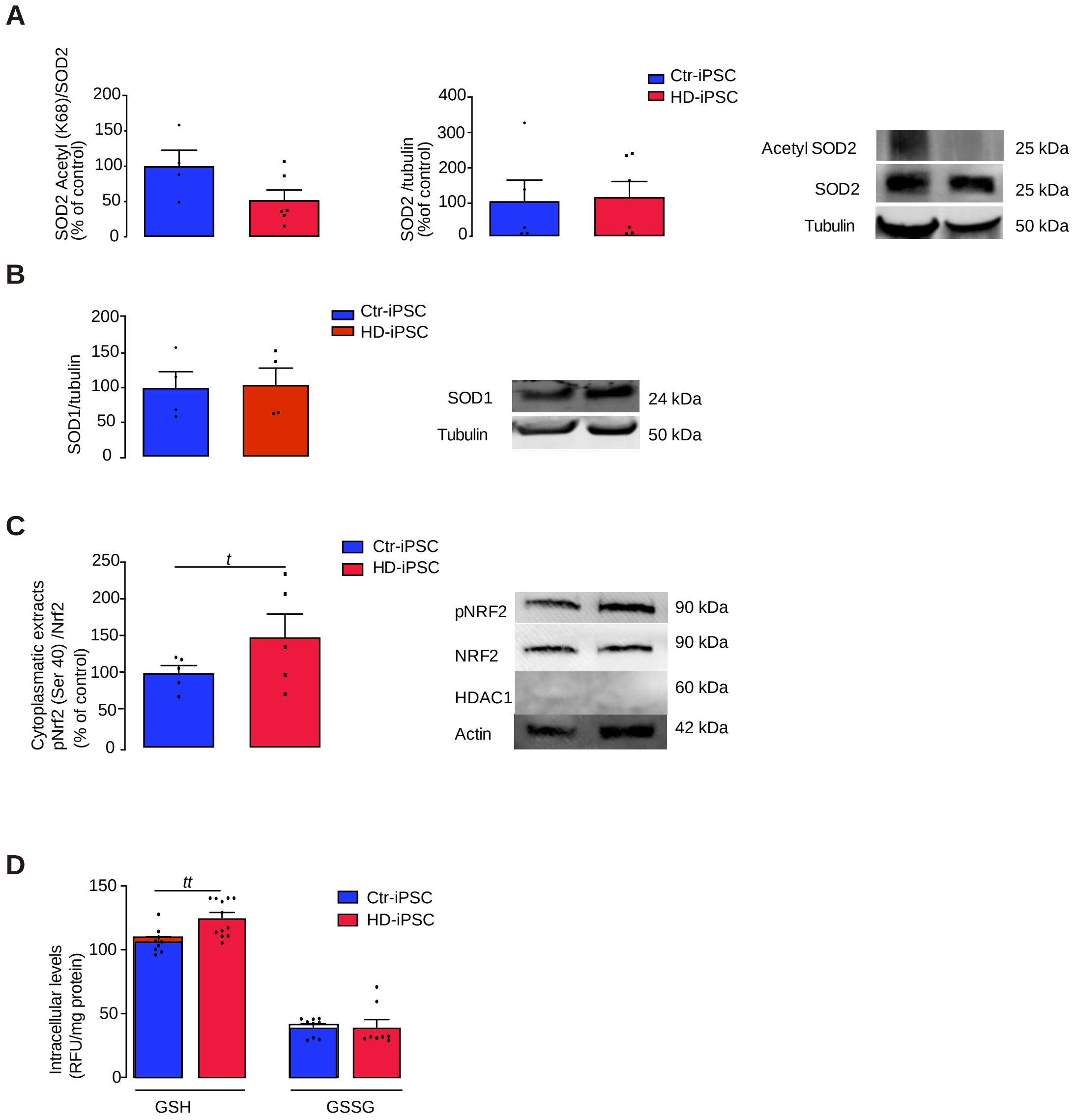

Figure S3. Antioxidant Responses to Oxidative Stress of iPSC. (**A-B**) SOD2 and 1 protein levels were analysed in iPSC and NSC cell lysates. Tubulin was used as a loading control. The results are expressed as the mean±S.E.M of at least 3 independent samples. (**C**) Phosphorylated Nrf2 protein levels in cytoplasmatic fractions in iPSC. Cytosolic enriched fraction was normalized to actin. The results are expressed as the mean±S.E.M of at least 3 independent samples. (**D**) Measurement of GSH and GSSG levels in iPSC. Values are the mean ± SEM of at least 3 independent samples. Statistical analyses were performed by Student's t-test $^{tt}p<0.01$.

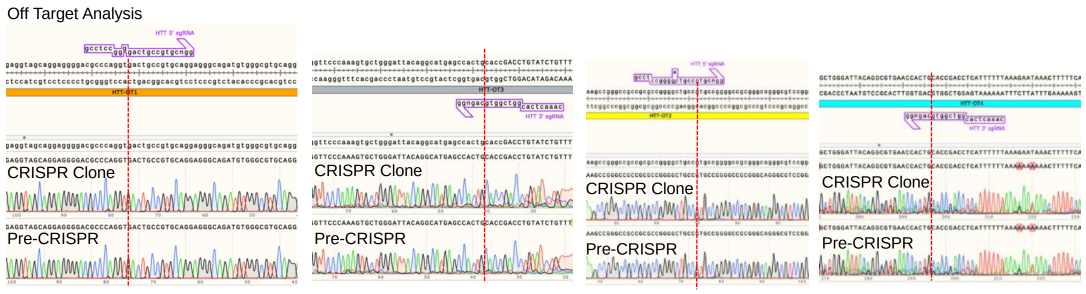

Figure S4. Potential off-target sites were identified using CasOffFinder (pmid: 24463181) and then ranked according to the number and location of mismatches using a Python script. All identified potential off-target sites featured at least 3 mismatches, including at least 1 within the PAM-proximal 12 nucleotides. Consistent with published observations according to which off-target mutations at such sites are exceedingly rare (pmid: 25425480, 24996165, 26212079, 24996167), we did not find any de novo mutations among the four tested most-likely off-target sites (genomic DNA PCR product Sanger sequencing data shown for the highest-scoring off-target loci of the 5' and 3' HTT sgRNAs).

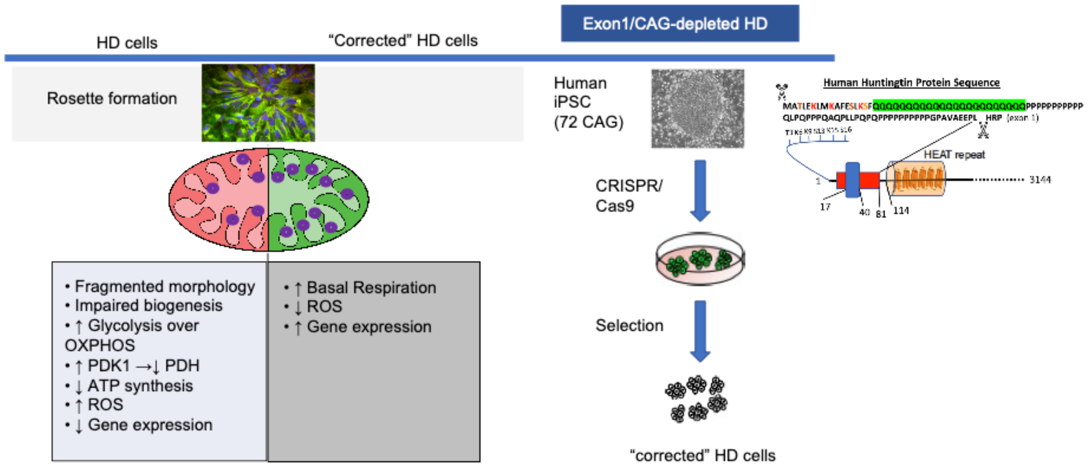

Figure S5. Schematic Model of Changes Occurring in HD and CAG Depleted Cells. HD-iPSC "corrected cells" can be successfully differentiated into NSC. CRISPR/Cas9 mediated excision of exon 1 of the *HTT* gene (scissor) reversed HD-associated impairment in mitochondrial respiration and downregulation of genes involved in mitochondrial biogenesis, resulting in lower production of mitochondrial reactive oxygen species. Mitochondria from HD-iPSC (red) exhibit lower mitochondrial respiration, metabolic deficit, reduced PGC-1 α and complex III subunit expression (purple dots) and activity, and were highly dependent on glycolysis, supported by pyruvate dehydrogenase (PDH) inactivation. Upon CRISPR/Cas9 deletion of CAG repeats HD-iPSC mitochondria (green) showed an improvement in basal respiration, a reduction of mitochondrial ROS and an increase in mRNA levels of complex III subunits (purple dots) supporting the rescue in mitochondrial function.

Supplemental Methods

Exosomes Isolation

Exosomes were isolated and purified by differential ultracentrifugation according to a described protocol (Thery et al., 2006). Briefly, after a filtration step (0.22 µm pore size) cells and tissue debris were eliminated by centrifugation at 10 000× g for 30 min. Supernatants were centrifuged at 100 000 × g (Type 45 Ti) for 70 min; after the supernatant was discard and pellet washed with PBS. An additional centrifugation at 110 000 × g (Type 45 Ti) for 70 min to vesicles isolation was performed. All centrifugations were performed at 4°C. Pellets were resuspended in PBS. Resuspended exosomes were used to Nanosight, TEM and mass spectrometry assays.

Immunocytochemistry

Cells were washed with warmed PBS 1x (137 mM NaCl, 2.7 mM KCl, 8 mM Na_2HPO_4, and 2 mM KH_2PO_4), followed by permeabilization with PHEM (5 mM HEPES, 60 mM PIPES, 10 mM EGTA, 2 mM $MgCl_2$, pH 7.0 with KOH)/0.1% Triton X-100 for 30 sec, and then fixed with 4% Paraformaldehyde (PFA)/PHEM (20 min, at RT). Cells were rehydrated with PBS/0.1% Triton X-100 (3 washes), blocked in 3% BSA/PBS (30 min) and incubated with the following primary antibody overnight at 4°C: anti-OCT4 (1:200), anti-SOX2 (1:200), anti-nestin (1:200), anti-OPA1 (1:100), anti-DRP1 (1:100), anti-MFN2 (1:500) and anti-Fis1 (1:100). Secondary antibodies and DAPI counterstain were applied for 1 h at room temperature. Confocal analysis was performed on a Zeiss LSM 710 confocal system (Carl Zeiss Microscopy).

Immunoblotting

Cells were lysed in lysis buffer (50 mM Tris-HCl pH 7.5, 0.1% Triton X-100, 2 mM EDTA, 2 mM EGTA, 50 mM NaF, 10 mM β-glycerophosphate, 5 mM sodium pyrophosphate, 1 mM sodium orthovanadate, 0.1% (v/v) β-mercaptoethanol, 250 µM PMSF, and 10 mg/ml aprotinin and leupeptin). For the isolation of nuclear and cytoplasmic fractions a Nuclear/Cytosol Fractionation Kit (BioVision, Inc.) was used according to manufacturer's instructions.

The samples were denatured with SDS sample buffer (50 mM Tris-HCl, pH 6.8, 2% sodium dodecyl sulphate, 5% glycerol, 0.01% bromophenol blue and 100 mM DTT), at 95ºC, for 5 min. Twenty-five µg or seventy µg protein were loaded in 6% or 12% gel, subjected to SDS-PAGE and electrophoretically transferred onto polyvinylidene difluoride (PVDF) Hybond-P membranes. Blots were blocked in 5% BSA/TBST buffer (20 mM Tris-HCl, 0.15 M NaCl, 0.1% Tween 20) and incubated with anti-HTT (D7F7-residues surrounding Pro1220) (1:1000) and polyglutamine stretch (1C2) (1:5000), anti-OCT4 (1:500), anti-DRP1 (1:500), anti-MFN2 (1:1000), anti-OPA1 (1:500), anti-phospho Nrf2 (S40) (1:500), anti-NRF2 (1:1000), anti-HDAC1 (1:1000), anti-GPx (1:1000), anti-GRed (1:2000), anti-acetyl SOD2 (1:250), anti-SOD2 (1:5000), anti-SOD1 (1:2000), anti-actin (1:5000) and anti-α-tubulin (1:1000). Membranes were further incubated with anti-rabbit and anti-mouse secondary antibodies (1:10000), for 90 min, at room temperature, and developed using ECF fluorescent reagent. Fluorescence signal was analysed using the QuantityOne software and the results were given as fluorescence intensity (INT)/mm^2. Data were presented as protein levels/α-tubulin or actin. Immunoreactive bands were visualized with VersaDoc Imaging System (BioRad®, Hercules, USA).

SWATH-MS analysis

Samples were filtrated using 0.2 µm PES membranes and the exosomes were isolated by ultracentrifugation at 100,000 xg for 70 min at 4°C. The pellets of exosomes were dissolved in 2x SDS-PAGE Sample Loading Buffer (Nzytech) aided by ultrasonication. Each sample was spiked with the same amount of a recombinant protein (MBP-GFP) to be used as internal standard (Anjo et al., 2018) and denatured at 95 °C for 5 min.

Ten microliters of each denatured sample were pooled to generate the proteome library (protein composition of each condition), and the remaining sample were used separately for SWATH-MS analysis to extract quantitative information. Samples were then alkylated with acrylamide and subjected to in-gel digestion by using the short-GeLC approach (Anjo et al., 2015) The formed peptides were subjected to SPE using OMIX tips with C18 stationary phase (Agilent Technologies) as recommended by the manufacture and then resuspended in LC-mobile phase (2% ACN and 0.1% FA).

Samples were analyzed on a TripleTOF™ 6600 System (Sciex®) using information-dependent acquisition (IDA) of pooled samples for protein identification and SWATH-MS acquisition of each individual sample for protein quantification (as presented in [2]). After the acquisition of each individual sample, those samples were combined in two pooled samples (iPSC and NSC pools) and four acquisitions of each pool were obtained to be used as technical replicates. Peptides were resolved by liquid chromatography (nanoLC Ultra 2D, Eksigent®) on a MicroLC ChromXP™ C18CL reverse phase column (300 µm × 15 cm, 3 µm particle size, 120 Å pore size; Eksigent) at 5µL/min with a multistep gradient: 0-2 min linear gradient from 2 to 5 %, 2-45 min linear gradient from 5 % to 30 % and, 45-46 min to 35 % of acetonitrile in 0.1 % FA and 5% DMSO. Peptides were eluted into the mass spectrometer using an electrospray ionization source (DuoSpray™ Source, ABSciex®) with a 50 µm internal diameter (ID) stainless steel emitter (NewObjective).

For information, dependent acquisition (IDA) experiments, the mass spectrometer was set to scanning full spectra (m/z 350-1250) for 250 ms, followed by up to 100 MS/MS scans (m/z 100–1500 from a dynamic accumulation time – minimum 30 ms for precursor above the intensity threshold of 2000 – in order to maintain a cycle time of 3.3 s). Candidate ions with a charge state between +2 and +5 and counts above a minimum threshold of 10 counts per second were isolated for fragmentation and one MS/MS spectra was collected before adding those ions to the exclusion list for 15 seconds (mass spectrometer operated by Analyst® TF 1.7, Sciex®). Rolling collision was used with a collision energy spread of 5.

For SWATH-MS based experiments, the mass spectrometer was operated in a looped product ion mode (Gillet et al., 2012) and the same chromatographic conditions used as in the IDA run described above. A set of 60 windows of variable width (containing a m/z of 1 for the window overlap) was constructed covering the precursor mass range of m/z 350-1250. The individual information [(including mass range, width, and collision energy spread (CES)] for each of the 60 SWATH windows (each product ion scan) can be found in Supplementary Table 1. A 50 ms survey scan (m/z 350-1250) was acquired at the beginning of each cycle for instrument calibration and SWATH MS/MS spectra were collected from m/z 100–1500 for 50 ms resulting in a cycle time of 3.3 s from the precursors ranging from m/z 350 to 1250. The collision energy (CE) applied to each m/z window was determined considering the appropriate CE for a +2 ion centered upon this window and the collision energy spread (CES) was also adapted to each m/z window (Supplementary Table 1).

Peptide identification and library generation were performed with ProteinPilot software (v5.0, Sciex®), using the following parameters: i) search against a database from

SwissProt composed by Homo Sapiens (release at June 2017) and MBP-GFP sequences ii) acrylamide alkylated cysteines as fixed modification; iii) trypsin as digestion type. An independent False Discovery Rate (FDR) analysis, using the target-decoy approach provided by ProteinPilot™, was used to assess the quality of the identifications and confident identifications were considered when identified proteins reached a 5% local FDR (Sennels et al., 2009; Tang et al., 2008). To increase the number of proteins in the SWATH library, protein identification was performed by combining the IDA files from the exosomes' pooled sample combined with the IDA files from a cellular extract.
Data processing was performed using SWATHTM processing plug-in for PeakViewTM (v2.0.01, ABSciex®). After retention time adjustment using the MBP-GFP peptides, up to 15 peptides, with up to 5 fragments each, were chosen per protein, and quantitation was attempted for all proteins in library file that were identified from ProteinPilotTM searches. Peptides' confidence threshold was determined based on a FDR analysis using the target-decoy approach and those that met the 1 % FDR threshold in at least three of the four technical replicates were retained, and the peak areas of the target fragment ions of those peptides were extracted across the experiments using an extracted-ion chromatogram (XIC) window of 4 minutes with 100 ppm XIC width. The levels of the proteins were estimated by summing all the filtered transitions from all the filtered peptides for a given protein and normalized to the internal standard.
Only proteins with coefficient of variation (CV) below 20% at the technical level were used for comparative analysis.

SUPPLEMENTARY TABLE 1 – SWATH-MS METHOD

	m/z range	Width (Da)	CES
**Window 1**	349.5-360.9	11.4	5
**Window 2**	359.9-375.2	15.3	5
**Window 3**	374.2-389.2	15	5
**Window 4**	388.2-402.2	14	5
**Window 5**	401.2-415.3	14.1	5
**Window 6**	414.3-427.4	13.1	5
**Window 7**	426.4-439.1	12.7	5
**Window 8**	438.1-449.9	11.8	5
**Window 9**	448.9-460.7	11.8	5
**Window 10**	459.7-471.1	11.4	5
**Window 11**	470.1-480.5	10.4	5
**Window 12**	479.5-490	10.5	5
**Window 13**	489-499	10	5
**Window 14**	498-508	10	5
**Window 15**	507-516.5	9.5	5
**Window 16**	515.5-525.1	9.6	5
**Window 17**	524.1-533.2	9.1	5
**Window 18**	532.2-540.8	8.6	5
**Window 19**	539.8-548.5	8.7	5
**Window 20**	547.5-555.7	8.2	5
**Window 21**	554.7-563.4	8.7	5
**Window 22**	562.4-570.6	8.2	5
**Window 23**	569.6-577.8	8.2	5

**Window 24**	576.8-585.4	8.6	5
**Window 25**	584.4-592.6	8.2	5
**Window 26**	591.6-600.3	8.7	5
**Window 27**	599.3-607.9	8.6	5
**Window 28**	606.9-615.6	8.7	5
**Window 29**	614.6-623.2	8.6	5
**Window 30**	622.2-630.9	8.7	5
**Window 31**	629.9-638.5	8.6	5
**Window 32**	637.5-646.2	8.7	5
**Window 33**	645.2-653.8	8.6	5
**Window 34**	652.8-661.5	8.7	5
**Window 35**	660.5-669.1	8.6	5
**Window 36**	668.1-677.2	9.1	5
**Window 37**	676.2-685.3	9.1	5
**Window 38**	684.3-693.9	9.6	5
**Window 39**	692.9-702.9	10	5
**Window 40**	701.9-711.9	10	5
**Window 41**	710.9-721.3	10.4	5
**Window 42**	720.3-731.2	10.9	5
**Window 43**	730.2-741.6	11.4	5
**Window 44**	740.6-752.4	11.8	5
**Window 45**	751.4-763.6	12.2	5
**Window 46**	762.6-775.8	13.2	5
**Window 47**	774.8-787.9	13.1	5
**Window 48**	786.9-800.5	13.6	8
**Window 49**	799.5-814.5	15	8
**Window 50**	813.5-829.3	15.8	8
**Window 51**	828.3-845.5	17.2	8
**Window 52**	844.5-865.3	20.8	8
**Window 53**	864.3-886.5	22.2	8
**Window 54**	885.5-911.2	25.7	8
**Window 55**	910.2-939.1	28.9	8
**Window 56**	938.1-972	33.9	8
**Window 57**	971-1008.4	37.4	10
**Window 58**	1007.4-1053.4	46	10
**Window 59**	1052.4-1120	67.6	10
**Window 60**	1119-1249.6	130.6	10

For each of the 60 SWATH windows (each of the 60 product ion scans), it is indicated the mass range covered in each window (m/Z range), the width of the window, and the collision energy spread (CES) used in each window.

DNA and RNA Extraction, cDNA and RT-qPCR

Genomic DNA was extracted using PureLink® Genomic DNA Kit, a silica membrane-based isolation procedure and RNA with the PureZOL® RNA Isolation Reagent. Briefly, cells were washed with cold PBS, lysed with proteinase K or PureZOL® and homogenized with the respective isolation kits according to manufacturer's instructions.

The RNA was precipitated with isopropyl alcohol and the final DNA and RNA pellets were resuspended in the kits elution buffers. The purified DNA and RNA was then quantified with NanoDropR spectrophotometer and considered as pure if the A260/A280 ratio was 1.8–2.0. Reverse transcription was performed on 1 µg of RNA with iScript™ cDNA Synthesis Kit. The reaction was performed as described: 5 min at 25ºC, 30 min at 42ºC, followed by 5 min at 85ºC and cooled at 4ºC.
The single-stranded cDNA concentration was determined, and 50 ng was used in each subsequent reaction. The cDNA was amplified using sequence-specific primers described in Table 2 and quantified relatively to tubulin or 18S.

SUPPLEMENTARY TABLE 2 - Primer sets used in qRT-PCR analysis.

**Target gene**	**forward (5'›3')**	**reverse (5'›3')**
PGC1-α	GTCACCACCCAAATCCTTAT	ATCTACTGCCTGGAGACCTT
TFAM	AGAGCAGTCTGGGAGTAGGG	TGCTGCATTTGTCCCGAGAT
CYC1	CACTGCGGGAAGGTCTCTAC	CATCATCAACATCTTGAGCCCC
UQCR10	TGGGCGTCATGTTCTTCGAG	CTCTACTCCACTGTCACCCC
MT-CYB	ACCCCCTAGGAATCACCTCC	GCCTAGGAGGTCTGGTGAGA
ND1	AAACCCGCCACATCTACCAT	GCCTAGGTTGAGGTTGACCA
COX3	CACCACCTGTCCAAAAAGGC	CTAGGGGATTTAGCGGGGTG
PDK1	GGACTTCGGGTCAGTGAATGC	CGCAGAAACATAAACGAGGT
PDP1	CCTCGTCGGGAAGAATCGTT	CCTCACAACTTTGGACGGGT
UCP2	AGCCCACGGATGTGGTAAAG	CTCTCGGGCAATGGTCTTGT
NRF2	CAGTCAGCGACGGAAAGAGT	AAGTGACTGAAACGTAGCCGA
HO-1	ACCCATGACACCAAGGACCAGA	GTGTAAGGACCCATCGGAGAAGC
GCLc	GTGGATGTGGACACCAGATG	GCGATAAACTCCCTCATCCA
hHTT	TAGGGCTGTCAATCATGCTG	CAAGGGAAGACCCAAGTGAG
18S	GAGGATGAGGTGGAACGTGT	TCTTCAGTCGCTCCACGTCT
TUBULIN	CCAGGGCTGTGTTTGTAGACC	CAATAGTGTAGTGTCCACGGGC

Real-time PCR (qPCR) was performed with iQ™ SYBR® Green Supermix on a CFX96 Touch™ Real-Time PCR Detection System. The amplification reaction mixture (10 µl) contained 2.5 µl of the cDNA template, 0.3 µM of each primer (10 µM) and 5 µl of iQ™ SYBR® Green Supermix. Q-PCR was performed according to manufacturer's protocol: enzyme activation at 95ºC for 3 minutes, followed by 40 cycles of denaturation at 95ºC for 15 seconds, annealing/extension for 45 seconds at the specif primer´s annealing temperature, and the melt curve from 55 to 95ºC for 5 seconds/step with a 0.5ºC of increment. Expression values were calculated using the ΔΔCt method. All PCR samples were run in technical triplicates, and the average Ct-values were used for calculations.

Measurement of mitochondrial and cellular superoxide anion and hydrogen peroxide

For MitoSOX® Red Mitochondrial Superoxide Indicator analysis, iPSC were detached by accutase, collected and allowed to rest for 30 min in KSR medium. Subsequently the cells were centrifuged 3 min, 1000 rpm (RT) and washed with Krebs medium (in mM: 135 NaCl, 5 KCl, 0.4 KH_2PO_4, 1.8 $CaCl_2$, 1 $MgSO_4$, 20 HEPES and 5.5 glucose, at pH 7.4). Cells were incubated at 37ºC with 5 µM MitoSOX® for 30 min, washed with Krebs and the resultant fluorescence analyzed on a Microplate Spectrofluorometer Gemini EM for 5 min after which the cells were treated with the stressor compounds antimycin A (1 µM AA) or pre-incubating the cells for 2 h with 300 nM 3-NP and fluorescence was measured for more 20 min. Results were obtained with excitation at 510 nm and emission at 580 nm. NSCs were cultured for 24 h at 37°C in 96-well assay plates coated with matrigel. Cells were incubated for 30 min with 5 µM MitoSOX® Red in HBSS and proceeded as described above. Results were obtained with excitation at 510 nm and emission at 580 nm.

NSCs were cultured for 24 h at 37°C in 96-well assay plates coated with matrigel. Cells were washed with HBSS (in mM: 137.9 NaCl, 1.3 $CaCl_2$, 0.5 $MgCl_2$-$6H_2O$ 0.4 $MgSO_4$-$7H_2O$, 5.3 KCl, 0.4 KH_2PO_4, 4.2 $NaHCO_3$, 0.3 Na_2HPO_4, 5.6 D-glucose, at pH 7.4) and incubated for 30 min with 5 µM MitoSOX® Red in HBSS and proceeded as described above.

Briefly, iPSCs and NSC were loaded with 10 µM of MitoPY1 probe for 30 minutes at 37ºC and 5% CO2. MitoPY1 fluorescence was obtained using a Microplate Spectrofluorometer Gemini EM. Basal levels were measured for 10-15 minutes followed by an acute stimulus of 3 µM of Myxothiazol A. MitoPY1 was excited at 503 nm and collection signal was at 530 nm.

To measure cellular H_2O_2 production, the Amplex® Red Hydrogen Peroxide/Peroxidase Assay Kit was used. The Amplex® Red reagent in contact with H_2O_2 produces resorufin, a red-fluorescent oxidation product with a excitation wavelengh at 571 nm and an emission at 585 nm. Briefly, iPSC were detached by trypsin, collected and allowed to rest for 30 min in KSR medium, as described previously. Media was then replaced by Krebs solution with 10 µM Amplex®Red reagent and 0.5 U/mL Horseradish (HRP) peroxidase. Fluorescence was measure for a total time of 40 minutes. For NSC assays, cells were cultured in 96-well assay plates coated with Geltrex in neural maintainance medium. Prior to the assay, cells were washed in HBSS and readings performed in the presence of 10 µM Amplex®Red reagent and 0.5 U/mL Horseradish (HRP) peroxidase. Resultant fluorescence was analyzed on a Microplate Spectrofluorometer Gemini EM. The results were calculated as RFU per 500.000 cells for iPSC or per mg of protein for NSC.

Enzymatic assays

For all enzymatic assays, cells were washed in PBS, lysed in the respective buffer, sonicated for 3 min and then centrifuged at 14000 rpm for 10 min, at 4ºC (Eppendorf Centrifuge 5417R). The resulting supernatant was used for the following methods, after protein quantification using the BioRad protein assay (Bradford method).

- **Measurement of superoxide dismutase (SOD) activity**

Determination of SOD enzymatic activity was performed accordingly to the SOD Assay Kit (Sigma-Aldrich). In order to measure SOD2 (Mn-SOD) activity, 2 mM potassium cyanide (KCN) (which inhibits SOD1 or Cu/Zn-SOD) was added. In brief, The superoxide dismutase (SOD) Assay Kit uses water-soluble tetrazolium salt, WST-1 (2-(4-iodophenyl)-3-(4-nitrophenyl)-5-(2,4-disulfophenyl)-2H tetrazolium, monosodium

salt) that produces a water-soluble formazan dye upon reduction by superoxide anion ($O_2^{\bullet-}$). The rate of the reduction by $O_2^{\bullet-}$ is linearly related to the xanthine oxidase (XO) activity, and is inhibited by SOD. Absorbance at 440 nm was proportional to the amount of $O_2^{\bullet-}$. Thus, decreased WST-1 formazan formation indicates increased SOD activity.

- **Measurement of GPx and GRed activities**

The enzymatic activity of GPx requires the addition of the co-factor GSH and the secondary enzyme GRed. The activity in samples were measured upon incubation with a phosphate buffer (0.25 M KH_2PO_4, 0.25 M KH_2PO_4, 0.5 mM EDTA, pH 7.0) containing 10 mM GSH and 1 unit of the enzyme GRed for 5 minutes in the dark at RT. Next, 12 mM tert-butyl hydroperoxide (t-BHP) was added and subsequent basal readings for 2 minutes obtained. The peroxidase reaction initiated after addition of 2.5 mM NADPH (in 1% $NaHCO_3$). For the measurement of the enzymatic activity of GRed, samples were incubated in phosphate buffer (0.2 M K_2HPO_4, 2 mM EDTA, pH 7.0) plus 2 mM of NADPH (in 1% $NaHCO_3$), followed by basal readings for 2 minutes. The reductase reaction was initiated with the addition of 20 mM GSSG. Absorbance was aquired at 340 nm using the Microplate Spectrophotometer SpectraMax Plus 384 (Molecular Devices, USA). Results were normalized for the protein contents.

Fluorimetric measurement of GSH and GSSG

GSH levels in the samples were measured based on its ability to form a fluorescent adduct in the presence of ortho-phthaldialdehyde (OPA) (ex: 350; em: 420nm) at a neutral pH. The samples were lysed in 15 mM Tris with 0.2% Triton X-100 pH 7.0. The GSH levels were measured afterward samples were added to OPA (5 mg/ml in methanol) and 167 mM NaH_2PO_4. After a 15-minute incubation in the dark, fluorescence was aquired with a Microplate Spectrofluorometer Gemini EM (Molecular Devices, USA).

Similarly, GSSG levels were measured based on the ability of GSSG to form a fluorescent adduct with OPA at pH 12. Incubation with N-ethylmaleimide (NEM) (25 mg/ml in methanol) forms a non-fluorescent adduct with GSH and thus prevents formation of GSH-OPA. Samples were prepared in the same buffer as for GSH. The samples were first incubated with NEM (5 mg NEM/ml methanol) for 5 minutes in the dark at RT. Next, the mixture was incubated with OPA (1 mg OPA/ml methanol) plus 100 mM NaOH for 15 minutes in the dark, at RT. Fluorescence was measured with excitation at 350 nm and emission at 420 nm in a Microplate Spectrofluorometer Gemini EM. In both cases, results are calculated as RFU normalised to the amount of protein in the sample.

Preparation of mitochondrial-enriched fractions from iPSC and NSC

In brief, the culture medium was removed and cells washed once with ice-cold PBS. Cells were scraped and collected in sucrose based buffer (250 mM sucrose, 20 mM HEPES, 10 mM KCl, 1.5 mM $MgCl_2$, 1 mM EDTA, 1 mM EGTA at pH 7.4) and homogenized on ice with a 2-ml glass/Teflon tissue grinder with a tight clearance with 100 slow up and down strokes. Subsequently, homogenized samples were subjected to centrifugation at 2300 rpm, for 12 min, at 4°C. The mitochondrial-enriched supernatant was collected immediately and kept at −80 °C until further analysis.

Measurement of mitochondrial respiratory chain complexes activities

The Mitochondrial-enriched fractions were assayed for the activity of mitochondrial complexes (Cx) I–IV by spectrophotometry.

- *Complex I: NADH–ubiquinone oxidoreductase assay*

Briefly, 800 µl K_2HPO_4 buffer (0.25 M, pH 7.2), fatty acid–free BSA (50 mg/ml), KCN (1 mM) and NADH (0.15 mM) were added to 1 ml cuvette. After sample quantification, 100 µg of protein was added and the final volume adjusted to 990 µl with distilled water. In parallel, a separate cuvette was prepared, containing the same amount of reagents and sample, but with the addition of rotenone (10 µM) (a selective inhibitor of mitochondrial complex I) solution (blank). To start the reaction, ubiquinone-1 (50 µM) was added and read for 5 min until the reaction was stopped with rotenone (10 µM). Cx-I activity, measured at 30°C, was determined at 340 nm by following the decrease in NADH absorbance due to ubiquinone (50 µM) reduction to ubiquinol (Ragan 1987). Cx-I activity, expressed in nanomoles/min/milligram of protein, was normalized for citrate synthase activity and corresponds to the rotenone (10 µM) sensitive rate.

- *Complex II: Succinate–ubiquinone oxidoreductase assay*

Briefly, 500 µl K_2HPO_4 buffer (0.1 M, pH 7.4), KCN (1 mM), succinate (20 mM), 6,6-dichlorophenolindophenol (DCPIP, 74 µM), EDTA-K2 (40 µg/ml) and rotenone (10 µM) were added in 1 ml cuvette. After sample quantification 150 µg of protein was added and the final volume adjusted to 990 µl with distilled water. In parallel, a separate cuvette was prepared, containing the same amount of reagents and sample but with the addition of thenoyltrifluoroacetone (TTFA) (5 mM) before solution (blank). To start the reaction decylubiquinone (500 µM) was added and read for 3 min until the reaction was stopped with TTFA (5 mM). Cx-II activity, measured at 37°C, was monitored at 600 nm by following the reduction of DCPIP (74 µM) by ubiquinol resulting from this reaction (Hatefi et al. 1978). Cx- II activity, expressed in nanomoles/min/milligram of protein, was normalized for citrate synthase activity and corresponds to complex II inhibitor TTFA, sensitive rate.

- *Complex III: Ubiquinol–cytochrome c reductase assay*

Briefly, 620 µl K_2HPO_4 buffer (0.056 M, pH 7.2), reduced cytochrome C (100 µM), KCN (1 mM), EDTA-K2 (1 mM, pH 7.2), $MgCl_2$-$6H_2O$ (1 mM) and rotenone (10 µM) were added to 1 ml cuvette. After sample quantification 50 µg of protein was added and the final volume adjusted to 998 µl with distilled water. In parallel, a separate cuvette was prepared, containing the same quantity of reagents and sample (blank). To start the reaction ubiquinol (20 µM) was added and read for 5 min. Cx-III activity was monitored at 550 nm by following the ubiquinol reduction of cytochrome C at 30°C. Cx-III activity, expressed in rate constant (k)/min/milligram of protein, was normalized for citrate synthase activity.

- *Complex IV: Cytochrome c oxidase assay*

Briefly, 100 µl K_2HPO_4 buffer (0.1 M, pH 7.0), dodecyl maltoside (1.25 mM), reduced cytochrome C (15 µM) and $K_3Fe(CN)_6$ (1 mM) were added to 1 ml cuvette, at 30ºC. After sample quantification, 100 µg of protein was added and the final volume adjusted to 1000 µl with distilled water. The reduced cytochrome C was prepared by mixing its oxidized form with ascorbate and then dialysed for 72 h against a 0.01 M phosphate buffer, pH 7.0, at 4°C. Cx-IV activity was monitored at 550 nm and the reaction was started after addition of reduced cytochrome C. The rate of cytochrome C oxidation was calculated as first-order reaction constant (k)/milligram of protein, and further normalized for citrate synthase activity.

- *Complex I+III: NADH cytochrome c oxidoreductase*

Briefly, 200 µl of Tris-HCl (0.05 M, pH 8.0) medium supplemented with BSA (5 mg/ml), oxidized cytochrome c (40 µM) and KCN (240 µM) were added to 1 ml cuvette and incubated for 4 min. After sample quantification, 50 µg of protein was added and the final volume adjusted to 990 µl with distilled water. In parallel, a separate cuvette was prepared, containing the same amount of reagents and sample, but with the addition of

(10 μM) rotenone solution (blank). To start the reaction NADH (800 μM) was added and read for 3 min until the reaction was stopped with rotenone (4 μM). The activity of complex I+III was measured at 30°C by the change in absorbance at 550 nm (ε = 19.1 $mM^{-1}cm^{-1}$) due to the reduction of oxidized ferricytochrome C (Hagopian et al., 2010). The activity of complexes I+III, expressed in nanomoles/min/milligram of protein, was normalized for citrate synthase activity.

- *Citrate synthase (CS) assay*

Briefly, 500 μl of Tris (200 mM, pH 8.0) with Triton X-100 (0.1% (vol/vol)), 5,5′-dithio-bis(2-nitrobenzoic acid) (DTNB) (200 μM), acetyl-CoA (200 μM) and 50 μg of sample were added in 1 ml cuvette. The volume was adjusted to 1000 μl with distilled water. The reaction started by adding oxaloacetic acid (100 μM). CS activity was performed at 412 nm following the reduction of DTNB (200 μM) in the presence of acetyl-CoA (200 μM) and oxaloacetate (100 μM) (Coore et al., 1971). CS activity was expressed in nanomoles/min/milligram of protein.

XF24 extracellular flux analyser

iPSC and NSC were seeded onto an XF24 Cell Culture Microplate coated with matrigel (Seahorse Bioscience) (Zhang et al., 2012) and incubated at 37ºC until getting confluent. One ml of XF Calibrant Solution was placed into each well of the sensor hydration microplate and the sensor cartridge placed onto the microplate. The plate was incubated with immersed sensors in a non-CO_2 incubator at 37°C for ~16 h (overnight). XF assay medium (DMEM) was supplemented with 17.5 mM glucose, 1 mM pyruvate and 2 mM glutamine, pH adjusted to 7.4, at 37°C. The cell medium was aspirated from each iPSC- or NSC–containing microplate without disturbing the cell monolayers attached on the well bottoms. Gently, each well was washed with 0.5 ml of XF assay medium at 37 °C. Then, 450 μl of XF assay medium at 37°C was added to each well and the plate placed into a non-CO_2 incubator at 37°C for 1 h. The final concentrations of the inhibitors added into injection ports were: A 1 μM oligomycin; B 1 μM FCCP; and C 1 μM rotenone plus 1 μM antimycin A.

Glycolysis can be measured directly by the XF Analyzer and displayed as the Extracellular Acidification Rate (ECAR). At first, cells were incubated in the glycolysis stress test medium (DMEM with 34.2 mM NaCl, 3 mg/l Phenol Red, 2 mM L-glutamine, pH 7.35) without glucose for 1 h, at 37°C. The port A injected a saturating concentration of glucose (10 mM) and catabolizes iPSC/NSC through the glycolytic pathway to pyruvate, producing ATP, NADH, water, and protons. The second injection was 1 μM oligomycin, which inhibits mitochondrial ATP production and thus shifts the energy production to glycolysis, with the subsequent increase in ECAR. The last injection was 100 mM 2-DG, which inhibited glycolysis through competitive binding to glucose hexokinase. The resulting decrease in ECAR further confirmed that the ECAR produced in the experiment was due to glycolysis.

For fatty acid oxidation measurements of iPSC KHB assay medium (containing 111 mM NaCl, 4.7 mM KCl, 2 mM $MgSO_4$, 1.2 mM Na_2HPO_4, 2.5 mM glucose) supplemented with 500 μM L-carnitine was used. The exogenous free fatty acid palmitate (200 μM), which can be internalized by intact cells, was added in both injection ports A, B (100 μM each) followed by the addition of etomoxir at port C. The component of OCR due to palmitate oxidation was determined by the addition of etomoxir, which diffuses into cells and is an inhibitor of carnitine palmitoyltransferase 1 (CPT1), an enzyme that transports long-chain fatty acids across the mitochondrial inner membrane, thereby effectively inhibiting fatty acid oxidation. The design study protocol in the XF24 Extracellular Flux Analyzer software for analyzing iPSC and NSC was to set the time to mix for 2 min, wait

for 2 min and measure for 4 min. OCR and ECAR were normalized to the amount of protein, determined by the Protein Assay reagent (Bio-Rad) in all experiments.

References

Anjo, S. I., Santa, C., & Manadas, B. (2015). Short GeLC-SWATH: A fast and reliable quantitative approach for proteomic screenings. *Proteomics*, *15*(4), 757-762.

Anjo, S. I., Simões, I., Castanheira, P., Grãos, M., & Manadas, B. (2018). A generic normalization method for proper quantification in untargeted proteomics screening. *bioRxiv*. https://doi.org/10.1101/307504

Coore, H. G., Denton, R. M., Martin, B. R., & Randle, P. J. (1971). Regulation of adipose tissue pyruvate dehydrogenase by insulin and other hormones. *Biochem J*, *125*(1), 115-127.

Gillet, L. C., Navarro, P., Tate, S., Röst, H., Selevsek, N., Reiter, L., Bonner, R., & Aebersold, R. (2012). Targeted data extraction of the MS/MS spectra generated by data-independent acquisition: a new concept for consistent and accurate proteome analysis. *Molecular & Cellular Proteomics*, *11*(6).

Hagopian, K., Weber, K. L., Hwee, D. T., Van Eenennaam, A. L., Lopez-Lluch, G., Villalba, J. M., Buron, I., Navas, P., German, J. B., Watkins, S. M., Chen, Y., Wei, A., McDonald, R. B., & Ramsey, J. J. (2010). Complex I-associated hydrogen peroxide production is decreased and electron transport chain enzyme activities are altered in n-3 enriched fat-1 mice [Research Support, N.I.H., Extramural]. *PLoS One*, *5*(9), e12696. https://doi.org/10.1371/journal.pone.0012696

Sennels, L., Bukowski-Wills, J. C., & Rappsilber, J. (2009). Improved results in proteomics by use of local and peptide-class specific false discovery rates. *BMC Bioinformatics*, *10*, 179. https://doi.org/10.1186/1471-2105-10-179

Tang, W. H., Shilov, I. V., & Seymour, S. L. (2008). Nonlinear fitting method for determining local false discovery rates from decoy database searches. *J Proteome Res*, *7*(9), 3661-3667. https://doi.org/10.1021/pr070492f

Thery, C., Amigorena, S., Raposo, G., & Clayton, A. (2006). Isolation and characterization of exosomes from cell culture supernatants and biological fluids. *Curr Protoc Cell Biol*, *Chapter 3*, Unit 3 22. https://doi.org/10.1002/0471143030.cb0322s30

Zhang, J., Nuebel, E., Wisidagama, D. R., Setoguchi, K., Hong, J. S., Van Horn, C. M., Imam, S. S., Vergnes, L., Malone, C. S., Koehler, C. M., & Teitell, M. A. (2012). Measuring energy metabolism in cultured cells, including human pluripotent stem cells and differentiated cells [Research Support, N.I.H., Intramural

Research Support, Non-U.S. Gov't]. *Nat Protoc*, *7*(6), 1068-1085. https://doi.org/10.1038/nprot.2012.048
